# Hair follicle epithelial stem cells contribute to interfollicular epidermis during homeostasis

**DOI:** 10.1172/jci.insight.193496

**Published:** 2025-07-08

**Authors:** Elnaz Ghotbi, Edem Tchegnon, Ze Yu, Tracey Shipman, Zhiguo Chen, Yumeng Zhang, Renee M. McKay, Chao Xing, Chung-Ping Liao, Lu Q. Le

**Affiliations:** 1 Department of Dermatology and; 2 McDermott Center for Human Growth and Development, University of Texas Southwestern Medical Center, Dallas, Texas, USA.; 3 Department of Dermatology, University of Virginia, Charlottesville, Virginia, USA.; 4 Department of Bioinformatics and; 5 O’Donnell School of Public Health, University of Texas Southwestern Medical Center, Dallas, Texas, USA.; 6 Graduate Institute of Medical Sciences, College of Medicine, Taipei Medical University, Taipei, Taiwan.

**Keywords:** Cell biology, Development, Stem Cells

## Abstract

Mammalian skin is a vital barrier with the epidermis serving as its protective outer layer, continually undergoing renewal. Given that loss of the epidermis or its barrier function is lethal for mammals, multiple stem cell populations likely exist for the interfollicular epidermis (IFE), enhancing evolutionary survival. Here, we demonstrate that transcription factor KROX20 marks a heterogeneous stem cell population in the upper and middle mouse hair follicle (HF), partially overlapping with known HF stem cell markers in those regions. Lineage tracing in mice using different reporter lines shows that *Krox20*-lineage cells migrate from the HF to the IFE, contributing to both basal and suprabasal layers during adulthood. Spatial transcriptomics data corroborate our findings. Depletion of epithelial *Krox20*-expressing cells leads to epidermal hyperplasia and a disruption of stratification during homeostasis. Our study highlights the contribution of hair follicle *Krox20*-lineage cells to the IFE and the regulation of epidermal homeostasis.

## Abstract 2

We identify a population of epithelial stem cells marked by the transcription factor *Krox20* whose lineage cells give rise to the interfollicular epidermis.

## Graphical abstract

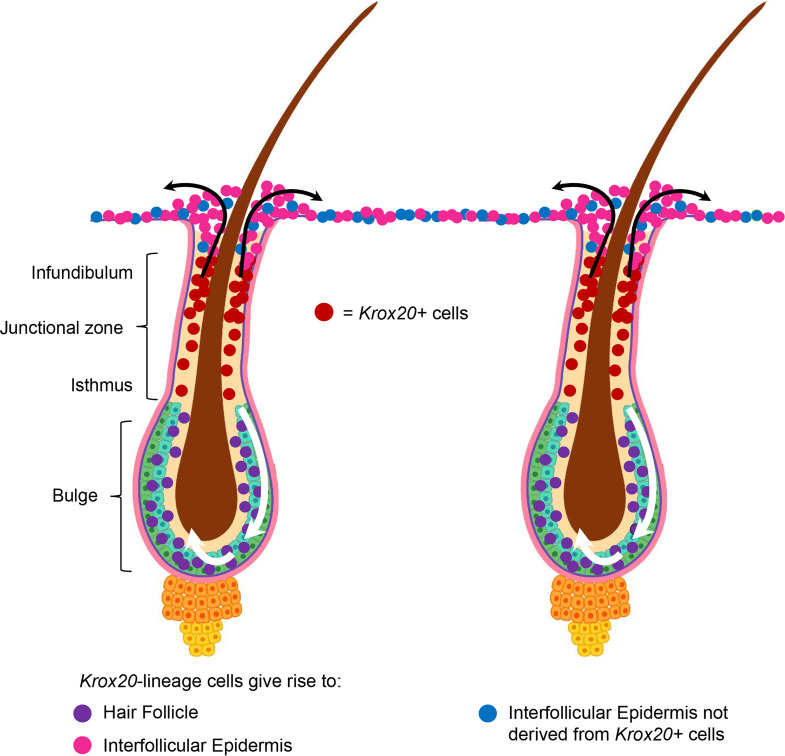

## Introduction

The mammalian skin functions as a crucial mechanical and biological safeguard. The skin’s epidermis comprises a basal layer with actively dividing cells and several suprabasal layers that undergo differentiation and enucleation as they migrate upward, ultimately leading to the formation of a cornified layer that is eventually shed from the skin surface (1). In maintaining homeostasis, the loss of epidermal cells during terminal differentiation must be precisely compensated for by the generation of new cells in the basal layer. One study suggests that the epidermis is sustained by a single proliferating committed progenitor cell population, where the balance between proliferation and differentiation results from random cell division fates (2). Conversely, another study indicates the presence of 2 distinct populations of epidermal progenitors in the basal layer of the interfollicular epidermis (IFE): a stem cell population and a progenitor cell population, each contributing differentially to the homeostasis of the epidermis (3). Prior research demonstrates that the epidermis and hair follicles (HFs) are maintained by local stem and progenitor cells in a compartmentalized manner during homeostasis (4). Stem cells within the epidermis are primarily located in the basal layer of the epithelium, identifiable by the basal keratins K5 and K14 (5). However, considering that the loss of the epidermis or its barrier function is incompatible with mammalian life (6), the existence of multiple stem cell populations for the IFE is likely, as this would enhance evolutionary survival. We previously reported *Krox20* (*Egr2*) as a marker for a population of epithelial cells in the HF that contribute to the formation of bulge (7) and hair shaft (8). Here, we demonstrate that *Krox20*-lineage cells also contribute to the IFE during homeostasis, introducing *Krox20*^+^ stem cells as a population contributing to both HF and IFE.

## Results

### Krox20-expressing cells are a heterogeneous population of epithelial stem cells in the upper HF.

KROX20 is a zinc finger transcription factor critical for the development and homeostasis of multiple tissues. Previous studies have documented the presence of *Krox20* in HFs during embryogenesis and early development, initiating at E14.5 (8, 9). Employing *Krox20-GFP* mice (which recapitulate endogenous *Krox20* expression; ref. 10) to visualize the expression of GFP, and using a KROX20 antibody in immunofluorescence assays, we demonstrated that *Krox20* is expressed predominantly in the HF infundibulum, spans from the upper to middle regions of the telogen and anagen HFs, and extends to the sebaceous glands (7) (Supplemental Figure 1, A and B; supplemental material available online with this article; https://doi.org/10.1172/jci.insight.193496DS1). This expression pattern within the HFs, coupled with its spatial colocalization with established stem cell markers such as *Lrig1* and *Lgr6* (4, 7), implies a substantial level of heterogeneity within this stem cell pool.

**Figure 1:**
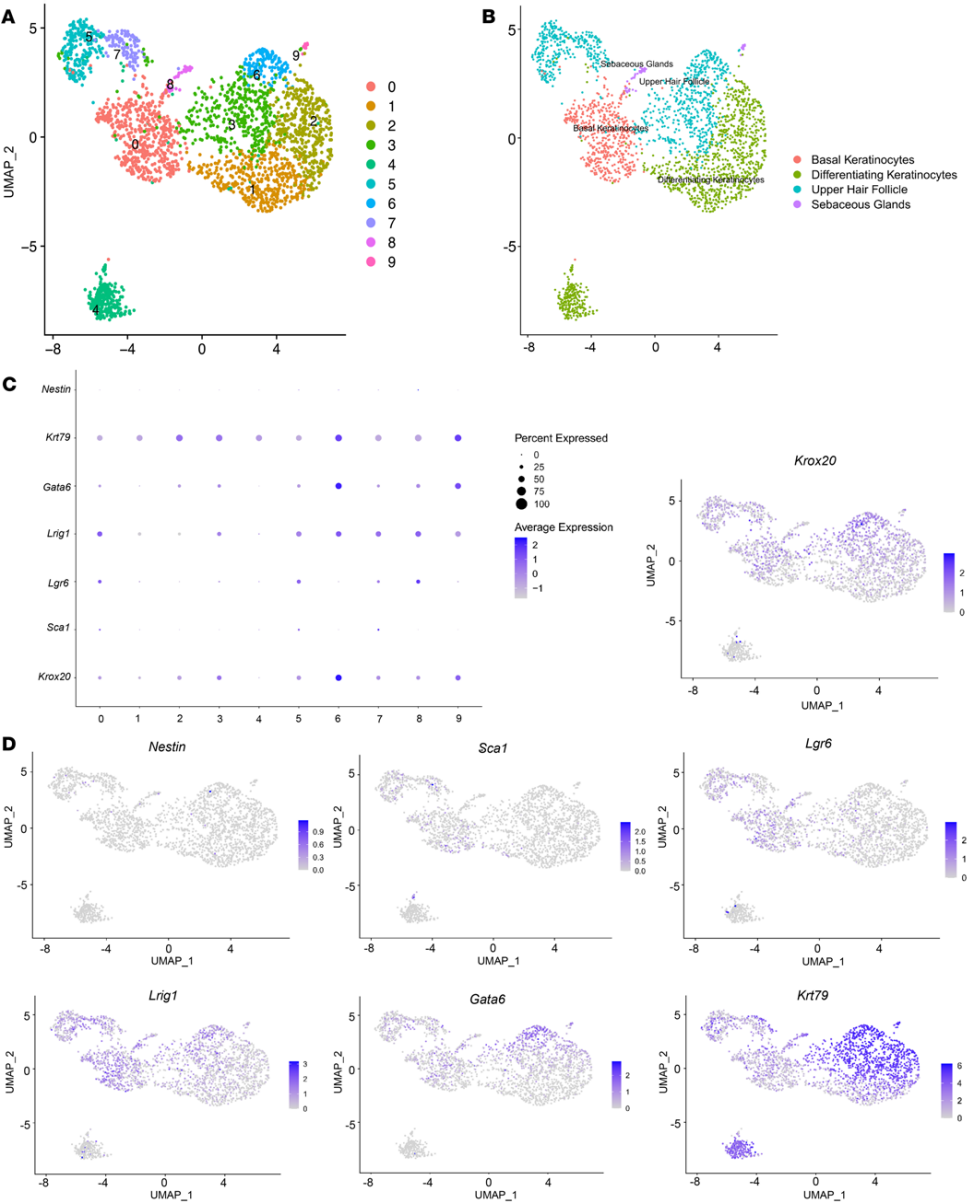
scRNA-Seq analysis on the epidermal cells of *Krox 20-GFP* P5 pups reveals cellular heterogeneity of *Krox20*-expressing cells. (**A**) UMAP representation of the transcriptomes of *Krox20*-expressing cells with annotated clusters (*n* = 5). Ten clusters of differentially expressed cells were identified. (**B**) Cell type annotations of the clusters identified. (**C**) Dot plot showing expression of *Nestin*, *Krt79*, *Gata6*, *Lrig1*, *Lgr6*, *Sca1*, (*Ly6a*), and *Krox20* in annotated clusters. (**D**) Expression of *Krox20*, *Nestin*, *Sca1*, *Lgr6*, *Lrig1*, *Gata6*, and *Krt79* visualized on UMAP. Color intensity from gray to blue shows the expression level for each gene.

To investigate this further, we conducted single-cell RNA-Seq (scRNA-Seq) on *Krox20*^+^ cells from epidermal cells of the skin of P5 *Krox20-GFP* mice. The analysis revealed 10 distinct clusters with differentially expressed genes, confirming the heterogeneity of *Krox20*^+^ cells (Figure 1A). Cell type annotations based on known gene markers identified basal and differentiating keratinocytes, upper HF, and sebaceous glands (Figure 1B). This distribution corresponded to the expression domain in the upper HF and sebaceous gland as shown in our immunofluorescence assays (Supplemental Figure 1, A and B) (7). As expected, *Krox20* expression overlapped with known upper and middle HF stem cell markers, particularly with *Lrig1*, *Lgr6*, *GATA6*, and *Krt79* and, to a lesser extent, *Sca1* (Figure 1, C and D). Additionally, *Nestin* expression was largely absent from these *Krox20*^+^ populations, with only a very small fraction of cells showing coexpression. Given that *Nestin* expression has been reported in the bulge region (11, 12), this minimal overlap confirms that *Krox20* is not expressed in the bulge (Figure 1, C and D).

### Krox20-lineage cells contribute to IFE during homeostasis.

To trace the lineage fate of *Krox20*^+^ cells, we generated an inducible *Krox20-CreERT*–knock-in mouse line as previously described (7). We used CRISPR/Cas9 to insert *CreERT* after exon 2 of *Krox20* following a P2A linker, thus keeping *Krox20* expression/function intact. We then bred *Krox20-CreERT* mice with *R26-tdTomato* reporter mice to generate *Krox20-CreERT; R26-tdTomato* mice. We excluded the potential for leakiness in this mouse line, as no *tdTomato* signal was observed in the absence of tamoxifen treatment (7) (Supplemental Figure 1C). However, a challenge in lineage tracing a heterogeneous population of cells is that performing the tracing at the clonal level, such as labeling a single cell and tracing its progeny, may not accurately represent the true capabilities or characteristics of the broader population of cells, even with multiple replicates.

**Figure 2:**
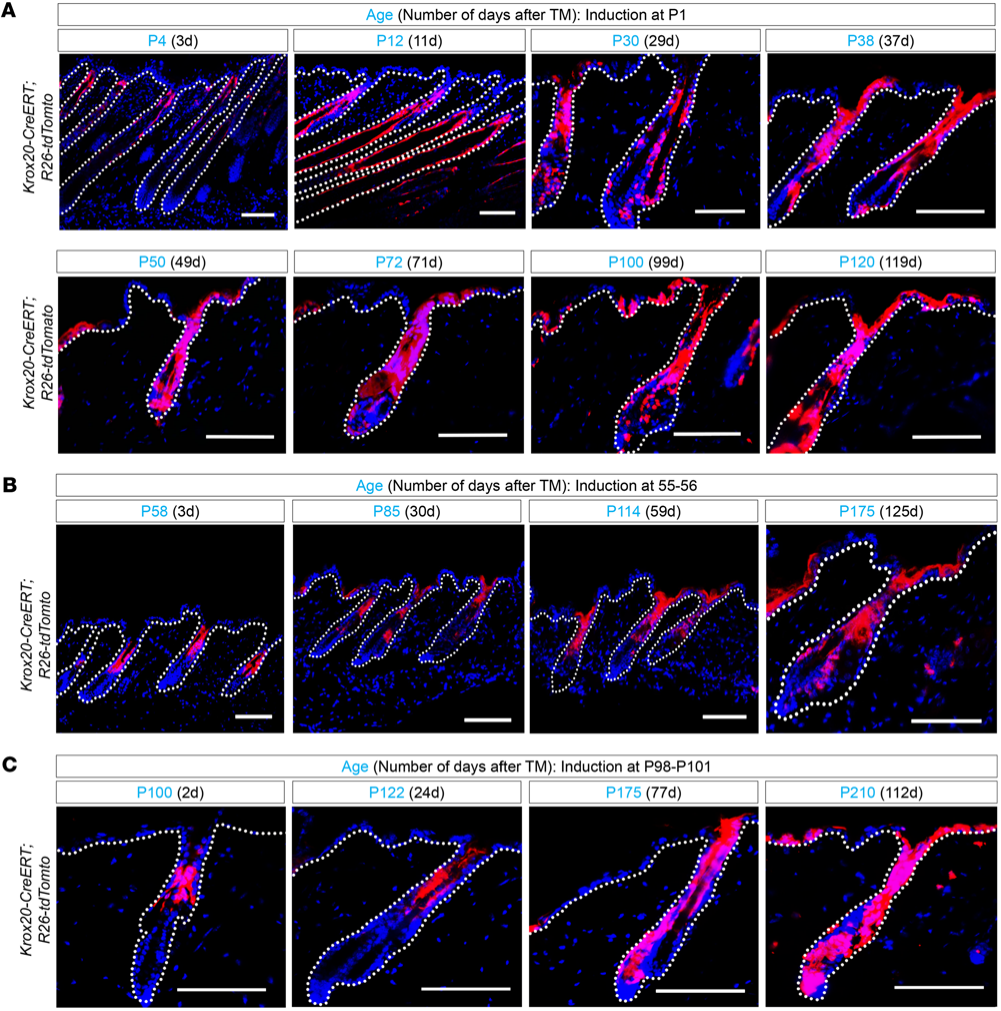
Lineage tracing in *Krox20-CreERT; R26-tdTomato* mice reveals contribution of *Krox20*-lineage cells to the IFE. (**A**–**C**) Lineage tracing initiated at P1 (**A**), P55 (**B**), and P98 (**C**) shows *Krox20*-lineage cells contribute to the HF and IFE over time. *n* ≥ 3. Scale bar: 100 μm.

In our lineage tracing analysis with *Krox20-CreERT; R26-tdTomato* mice, we induced labeling to the extent that we observed most *Krox20*-expressing cells labeled at the onset of our lineage tracing analysis (Figure 2, A and B). This approach ensures that the full range of behaviors/fates that are possible in the entire population of *Krox20*-expressing cells will be captured. Therefore, mice were induced with 40 μg 4-hydroxytamoxifen at P1, and their skin was subsequently examined at different time points, spanning from 3 to 119 days after induction (Figure 2A). At P4, 3 days after induction, *Krox20*-lineage cells were confined to the expression domain of *Krox20* in the upper and middle regions of the anagen HF. However, *Krox20*-lineage cells were detected in the bulge and IFE by P30, and the contribution of lineage-traced cells to these regions increased in subsequent hair cycles (Figure 2A).

When tamoxifen induction was initiated later in development at 8 weeks of age, which corresponds to the second long and synchronous telogen phase of the hair cycle (P55–P56), and HFs were examined 3 days later, the *tdTomato*-expressing cells remained confined to the upper and middle telogen HFs (Figure 2B; P58), closely resembling the “live” expression pattern observed with *Krox20*-GFP (Supplemental Figure 1A). The lineage cells were detectable in the IFE by 30 days (Figure 2B; P85) and were expanding by 59 days after gavage (Figure 2B; P114). By 18 weeks after induction, the lineage cells were detected in both the bulge and IFE (Figure 2B; P175). When tamoxifen induction was performed following multiple hair cycles at P98–P101, the *tdTomato*-expressing cells remained confined to the upper and middle telogen HFs when examined 2 days after induction (Figure 2C; P100). Subsequently, these cells extended down the HF, partially reaching the bulge region by 11 weeks (Figure 2C; P175). By 16 weeks after induction, the lineage cells expanded their occupancy of the bulge while also contributing to the IFE (Figure 2C; P210). These findings provide conclusive evidence that *Krox20*-lineage cells continue to populate the postnatal IFE perinatally and throughout adulthood. The quantification of *Krox20*-lineage cells labeled by *tdTomato* in the IFE in *Krox20-CreERT; R26-tdTomato* mice induced at P1, P55, and P98 is shown in Supplemental Figure 2, A–C, respectively.

**Figure 3:**
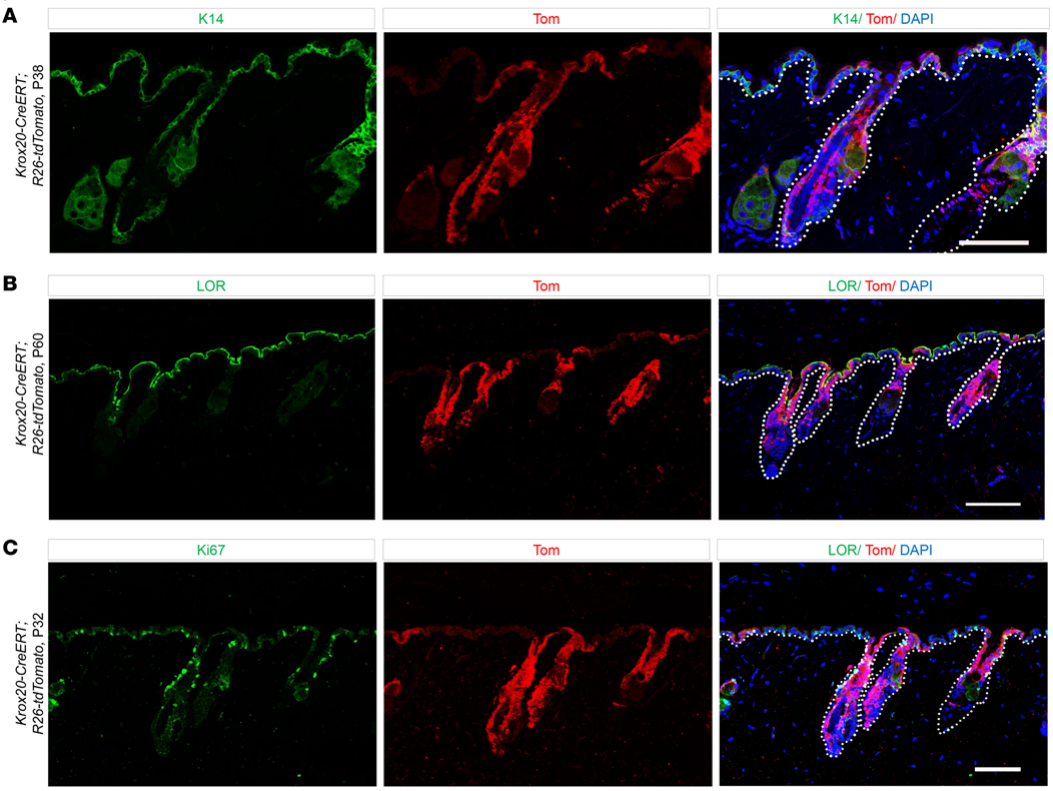
*Krox20*-lineage cells overlap with IFE markers. (**A**–**C**) Colocalization analysis demonstrates the detection of *tdTomato*-labeled (Tom = *tdTomato*) *Krox20*-lineage cells in relation to the K14 marker of basal layer of IFE (**A**), the loricrin (LOR) marker of suprabasal granular layer (**B**), and the Ki67 marker of proliferating cells (**C**) in telogen HFs at P38, P60, and P32, respectively, induced at P1. *n* ≥ 4. Scale bar: 100 μm.

Immunostaining of *tdTomato* together with IFE markers, including K14 for the basal layer and loricrin (LOR) for the suprabasal layers of IFE, revealed the colocalization of *Krox20*-lineage cells with both basal and suprabasal layers of IFE (Figure 3, A and B). This suggests the migration of *Krox20*-lineage cells from the HF to the basal layer of IFE, where they subsequently undergo differentiation, migrate to the suprabasal granular layer, and express Lor. Furthermore, *Krox20*-lineage cells colocalize with the Ki67 proliferation marker in the basal keratinocytes of IFE and HFs, indicating that *Krox20*-lineage cells contain actively cycling cells (Figure 3C). Lineage tracing using *Krox20-Cre; R26-tdTomato* mice yielded similar findings, with *tdTomato* labeling the upper part of the HFs during the perinatal period (late gestation E18.5 and early postnatal P5), before migrating to the IFE by P30 (Supplemental Figure 3, A and B). Our lineage tracing findings provide strong evidence that *Krox20*-lineage cells in the IFE originate from the HF. Given that epithelial *Krox20*^+^ cells represent a subgroup of embryonic *K14*^+^ cells (8), this finding suggests that *Krox20*-lineage cells may reactivate *K14* expression once they migrate to the basal layer of the IFE to contribute to skin homeostasis.

### Comprehensive validation of lineage tracing interpretation through multiple alternative approaches.

Our lineage tracing studies revealed that *Krox20*-expressing cells within the HF contribute to the IFE. However, interpreting such data requires caution, as previous studies have shown discrepancies in marker and/or reporter expression. For instance, *Lgr6*^+^ cells, initially believed to be a stem cell marker of the HF isthmus, were reported to contribute to the formation of the HF, sebaceous gland, and IFE (13). However, later studies reported the expression of *Lgr6* in IFE and sebaceous glands, questioning the validity of the lineage tracing results in the initial study (4, 14). Additionally, another study demonstrated the detection of YFP in the basal layer of the IFE 1 week after induction using *Involucrin-CreER; R26-YFP* mice, despite the absence of involucrin protein expression in the immunostaining assay (3). Therefore, there remains the possibility that very low levels of *Krox20*, and consequently *CreERT*, are expressed in the IFE but fall below the detection capability of our current methods, leading to the detection of *tdTomato* in the IFE rather than the migration of *Krox20*-lineage cells.

To address this concern and confirm the interpretation of our lineage tracing data, we took a multipronged approach to rule out this possibility. Utilizing the tamoxifen-inducible *Krox20-CreERT* line for lineage tracing adds a layer of confidence that the labeling of the IFE, which begins long after tamoxifen administration (a month after the tamoxifen induction at P1 and 112 days after the induction at P98), represents the migration of *Krox20*-lineage cells and is not due to the expression of *CreERT* in these regions.

However, even with inducible *CreERT* lines, a low level of leakiness/ background activity of basal *CreERT* is possible, which could result in reporter activation occurring independently of tamoxifen induction (15). In this regard, reporter lines such as *mTmG* and *R26-YFP* were shown to be more faithful reporters, exhibiting a higher recombination threshold for basal *CreERT* leakage compared with *R26-tdTomato* (15). While the possibility of background activity of *CreERT* in our *Krox20-CreERT; R26-tdTomato* was already excluded (7) (Supplemental Figure 1, C and D), to confirm the interpretation of our lineage tracing findings obtained using the *R26-tdTomato* reporter line, we repeated these experiments using *R26-YFP* reporter mice. These mice were administered a single dose of 4-hydroxytamoxifen (at P3) and subsequently underwent skin analysis at 2 different time points: 14 days and 43 days after induction. At 2 weeks after tamoxifen treatment, the *Krox20*-lineage cells in the *Krox20-CreERT; R26-YFP* mice were predominantly localized to the upper HF (Supplemental Figure 4A). However, by P43, the *Krox20*-lineage cells were detected at the bulge and IFE regions (Supplemental Figure 4B). These findings confirm the results obtained from the lineage tracing experiment conducted with *Krox20-CreERT; R26-tdTomato* mice. Of note, all lineage tracings conducted with *Krox20-CreERT* and the 2 different reporter lines (*R26-YFP* and *R26-tdTomato*) consistently demonstrated that the labeling of the IFE initiates/extends from the HF “mouth.” From this point, the labeling extended contiguously, marking the IFE. This labeling pattern is consistent and reproducibly observed, providing a robust basis for our observation regarding the HF origin of a specific subset of stem cells within the IFE.

We further evaluated the contribution of *Krox20*-lineage cells to IFE when lineage tracing is initiated at various yet overlapping ages, using the inducible *Krox20-CreERT; R26-tdTomato*. When tamoxifen induction was performed perinatally (at P1), *Krox20*-lineage cells were observed in the IFE by P30 (Figure 2A). However, the contribution of these lineage-traced cells to these regions increased in subsequent hair cycles (Figure 2A). When tamoxifen induction was performed between P98 and P101, *Krox20*-lineage cells were primarily detected in the upper HF at 2-days after induction, with virtually no presence in the bulge and IFE (Figure 2C). Conversely, in mice at a similar age (P100), but induced at P1, *Krox20*-lineage cells were already detected at bulge and IFE regions (Figure 2A). The difference between these results demonstrates that the *tdTomato* signal detected in the bulge and IFE reflects the progeny of *Krox20*^+^ cells, rather than representing live *Krox20* expression in these regions. Conducting the lineage tracing in *n* ≥ 4 replicates and consistently detecting *tdTomato* signal within the IFE at approximately the same time points among the replicates strengthens our claim that *Krox20*-lineage cells in the IFE migrated upward from the HF.

Previous research has revealed fate plasticity for various epidermal stem cells in response to injury or during wound healing (16, 17). To exclude the possibility of microwounds or small abrasions from mouse scratches, influencing the migration of *Krox20*-lineage cells to IFE, female and male mice — housed separately in cages without cohabitation — were utilized for a number of replicates for lineage tracing assays in this study.

**Figure 4:**
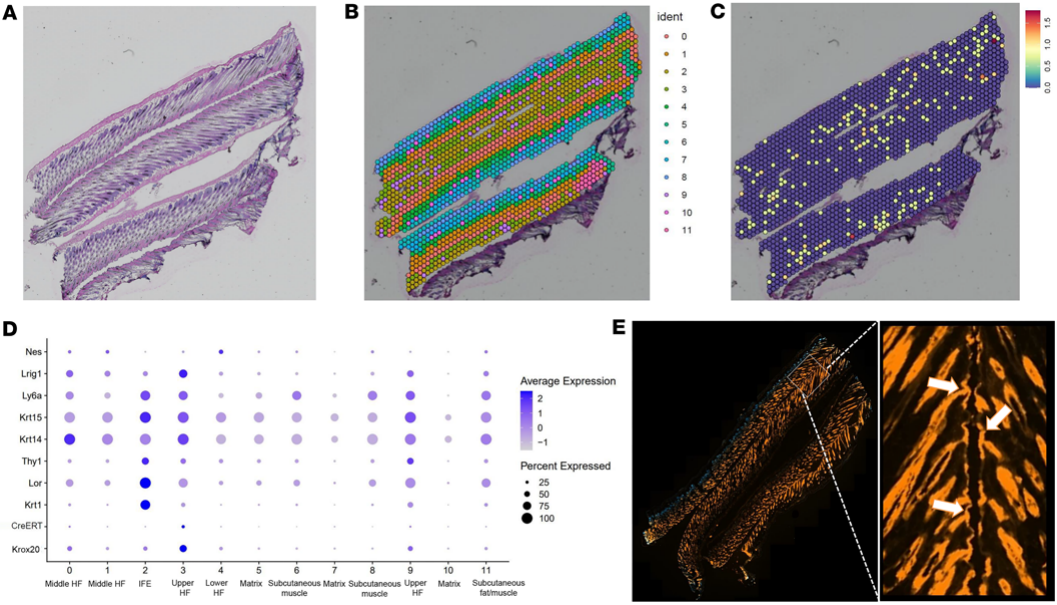
Spatial transcriptomic analysis of the dorsal skin of *Krox20-CreERT; R26-tdTomato* mice at P38. (**A** and **B**) Identification of 12 clusters of cells with differentially expressed genes, with cluster 1 representing the IFE. H&E-stained cross-sections of the skin, each section representing 1 mouse (*n* = 3, **A**). Note that the fourth section was excluded from analysis due to distorted structure. Spatial dim plots for all clusters (**B**). (**C**) Spatial feature plot for expression of *CreERT* is presented. Color intensity from blue to red shows the expression level for each gene. (**D**) Dot plot illustrating the expression levels of *Krox20*, *CreERT*, the marker of the upper and middle HF *Lrig1*, the marker of bulge stem cells *Nestin* (Nes), and IFE markers (*K14*, *K15*, *K1*, *Thy1*, *Sca1*, and *loricrin*) in the different gene clusters along with cluster annotations. (**E**) Live expression of *tdTomato* from the same 4 skin samples presented in **A**–**C**. White arrows point to the labeled HF mouth or IFE. Original magnification, ×20.

To further ensure the accuracy of our lineage tracing results and to avoid potential overinterpretation due to *CreERT* expression in the IFE, we employed spatial transcriptomics to analyze the precise expression pattern of *Krox20* and *CreERT*. Dorsal skin samples were collected from *Krox20-CreERT; R26-tdTomato* mice at P38 (*n* = 3), induced at P2, with each mouse corresponding to 1 skin section per slice (Figure 4A). This approach identified 12 clusters of cells with differentially expressed genes (Figure 4B). Cluster 2 was identified as the IFE (Figure 4B); however, due to the resolution limitations of the technique, we were unable to distinguish between basal and suprabasal layers within the IFE as well as the bulge area from the nearby middle HF regions. The spatial feature plot of *CreERT* expression in different regions of HF and skin is shown in Figure 4C. The dot plot in Figure 4D illustrates the expression of *CreERT* and *Krox20*, along with other IFE markers (*K14*, *K15*, *K1*, *Sca1/Ly6a*, *Thy1*, and *loricrin*), *Lrig1* (a marker of the upper HF), and *Nestin*, (11, 12) in different clusters. Our analysis of *Krox20* expression in the IFE revealed that approximately 35% of cells in cluster 2 exhibited *Krox20* expression (scaled average expression of 0.43) (Figure 4D). The dot plot demonstrates a correlation between *Krox20* and *CreERT* expression across various clusters, with *CreERT* expression levels being lower than those of *Krox20* (Figure 4D). This aligns with the immunostainings performed with CRE antibody on the skin sections from *Krox20-CreERT; R26-tdTomato* mice induced at P1 and analyzed at P22 (Supplemental Figure 5A), and P37 (Supplemental Figure 5B); these analyses showed low intensity of CRE signals. Notably, no CRE staining was detected in the IFE at P37, the time point when the IFE was labeled by *tdTomato* (Supplemental Figure 5B).

Importantly, *Nestin* expression was detected in clusters corresponding to lower and mid-HF regions. While *Krox20* and *Nestin* showed limited coexpression, their spatial expression patterns were largely distinct (Figure 4D), suggesting that they mark separate cell populations within the follicle consistent with our scRNA-Seq data in Figure 1, C and D.

Previous studies have suggested decreased protein expression at the position downstream of P2A in bicistronic constructs (18). Therefore, the lower expression level of *CreERT* compared with *Krox20* is likely attributed to the design of the *Krox20-CreERT* line, which contains *CreERT* downstream of a P2A linker (7). While *tdTomato* labeling was observed extending from the HF mouth in the IFE at this age (Figure 4E; P38), the dot plot analysis indicated that *CreERT* expression was restricted to the main expression domain of *Krox20*, which is in the upper HF, and was absent from other regions of the epidermis, including the IFE (scaled average expression of 0.28) (Figure 4D). This observation rules out the possibility of unintended activation of *tdTomato* due to the background activity of *CreERT* and provides strong evidence that, while *Krox20* is expressed at low levels and in a subset of cells in the IFE, *tdTomato* labeling of the IFE represents *Krox20*-lineage cells that have migrated from the HF upward to contribute to the IFE.

The conclusion from our lineage tracing studies is directly supported by a recent study demonstrating that cells from the upper HF progressively integrate into the IFE and modulate skin barrier function (19). This independent validation strengthens our findings and confirms the HF as a cellular reservoir for the IFE, reinforcing the validity of our observations.

### Krox20-lineage cells do not contribute to volar skin.

**Figure 5:**
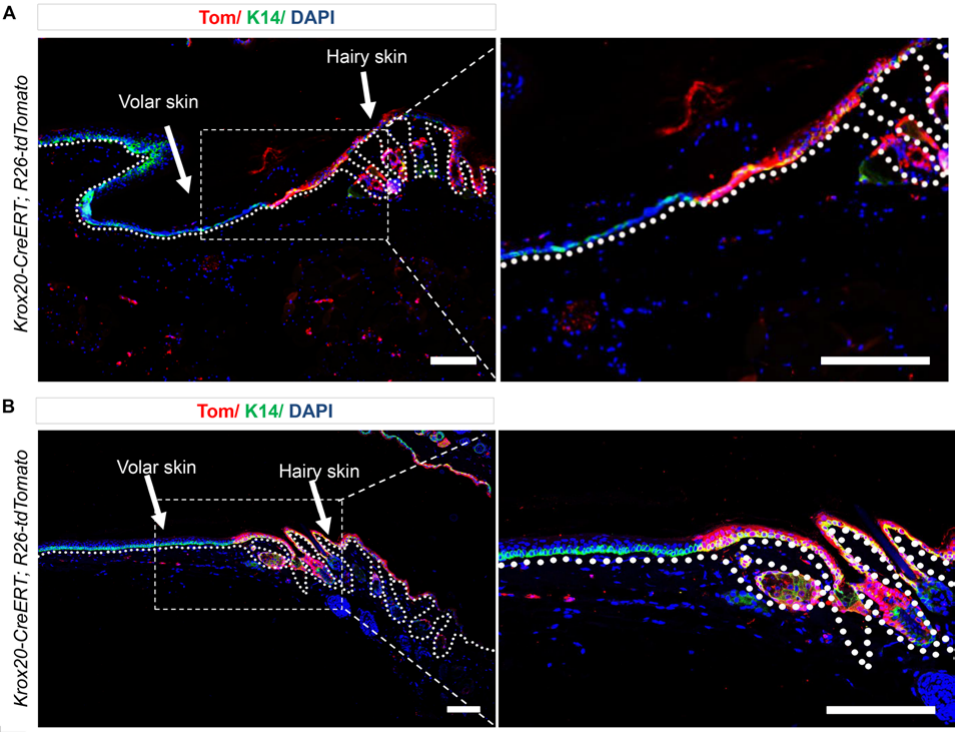
*Krox20* is not expressed in the volar epithelium. (**A** and **B**) Lineage tracing in 11-month-old *Krox20-CreERT; R26-tdTomato* mice, induced at P1 (**A**) — and P158 mice, induced at P55 (**B**) — shows the absence of *Krox20*-lineage cells in the volar epithelium of palmoplantar skin. *n* = 2. Scale bar: 100 μm.

In both humans and mice, the majority of skin surfaces are associated with HFs, with major exceptions being the hairless and thickened skin of the palm and sole (volar skin). Since *Krox20*^+^ cells mark a population of epidermal stem cells within the HF, we performed lineage tracing in volar skin of *Krox20-CreERT; R26-tdTomato* mice, inducing at P1 and analyzed at 11 months (Figure 5A) as well as inducing at P55 and analyzing at P158, to determine whether *Krox20*-lineage cells are detected in volar epidermis. We observed a distinct discontinuation of *Krox20*-lineage cells at the junction of hairy and hairless IFE (Figure 5, A and B), suggesting that the *tdTomato* labeling of the IFE has a HF origin. These findings show that *Krox20*^+^ cells exclusively serve as the stem cells for hairy epidermis and suggest the existence of other stem cell populations within the IFE that are responsible for maintaining the homeostasis of volar epithelia.

### Krox20^+^ cells are indispensable for skin homeostasis.

**Figure 6:**
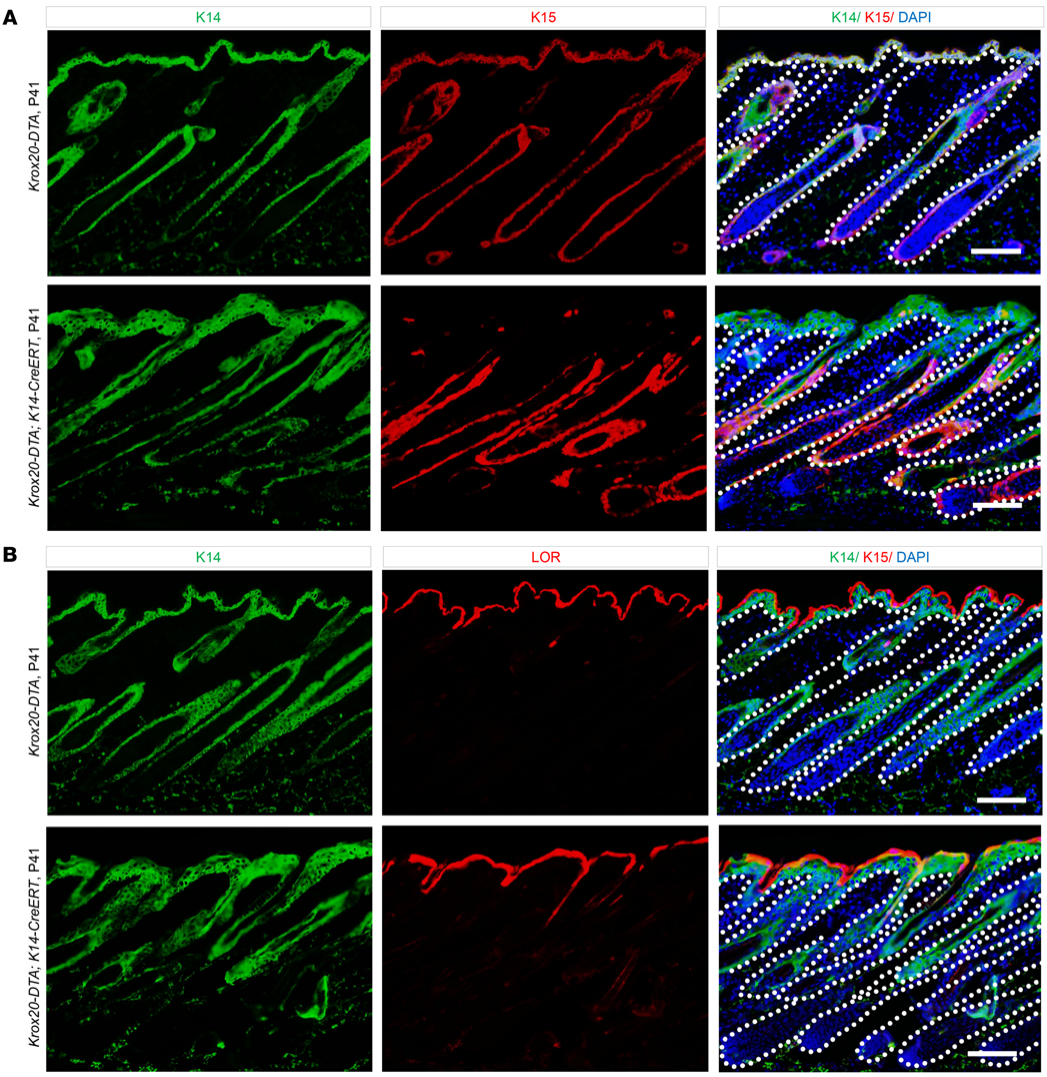
*Krox20^+^* cells are indispensable for skin homeostasis. (**A** and **B**) Analysis of the skin of *Krox20-DTA; K14-CreERT* mice at P41 induced at P32 shows the disruption of skin homeostasis and stratification as indicated by the absence of K15 expression in the IFE (**A**), and overlap of the suprabasal marker LOR with the K14 basal marker in the IFE (**B**). *n* = 3. Scale bar: 100 μm.

To determine the effect of ablating *Krox20*^+^ cells on the skin during homeostasis, we eliminated *Krox20*-expressing cells in the skin by breeding mice with the *Krox20-lox-Stop-lox-DTA* (*Krox20-DTA*) knock-in allele (10) with a *K14-CreERT* line (20), generating *Krox20-DTA; K14-CreERT* mice. In this model, *Krox20*-expressing cells of the *K14* lineage express diphtheria toxin A (DTA) and are ablated upon gavage with tamoxifen. Using this model, we ablated epithelial *Krox20*-expressing cells in the skin of mice at P32, which approximately corresponds to the time point when *Krox20*-lineage cells begin migrating to the IFE (Figure 6, A and B). The analysis of the skin 9 days after the ablation at P32 (P41), showed that depletion of *Krox20*-expressing cells resulted in hyperplasia of the epidermis (Figure 6, A and B). To determine the cause of epidermal hyperplasia in these mice, we first analyzed the basal cell marker K14. Strikingly, K14 was expressed in the entire epidermis of mutant mice, and the K14^+^ layer was markedly thicker. We then explored the effect of *Krox20*^+^ cell deletion on the bulge, as well as the basal and suprabasal layers of IFE by immunostaining for K15, a marker for both the bulge and basal layer of stratified epithelium (21, 22), and LOR, a marker of suprabasal granular cells. Notably, the elimination of epithelial *Krox20*^+^ cells led to the loss of *K15*^+^ epidermal stem cells in IFE (Figure 6A). In control epidermis, the LOR layer exists as a discrete layer superficial to the K14^+^ basal layer (Figure 6B). However, in the absence of *Krox20*^+^ cells, the LOR layer overlapped with the K14 layer within the suprabasal layer (Figure 6B). These findings demonstrate that the loss of *Krox20*^+^ cells disrupts normal epidermal stratification and suggest an essential role for *Krox20*-expressing cells in maintaining skin homeostasis in adulthood.

A potential contributing factor to the epidermal hyperplasia observed in our model may be an inflammatory response triggered by HF degeneration. To explore this possibility, we conducted immunofluorescence analyses targeting specific immune cell markers, CD3 for T cells, CD4 for Th cells, CD8 for cytotoxic T cells, and IBA1 for macrophages (Supplemental Figure 6, A–D). Among these, only IBA1 staining showed a notable increase, indicating enhanced macrophage presence in the dermis of *Krox20-DTA; K14-CreERT* mice. In contrast, we found no substantial evidence of infiltration by other immune cell types. These results point toward a localized macrophage response rather than a broad immune cell recruitment. Whether this macrophage accumulation is a driving factor in the development of hyperplasia or a downstream effect of tissue disruption remains an open question that warrants further investigation.

### KROX20 expression regulates cell fate determination by preventing epithelial differentiation.

Given the epidermal hyperplasia phenotype observed as the result of ablating *Krox20*^+^ cells in *K14*-expressing cells, we investigated the functional role of KROX20 in epithelial cells by overexpressing *Krox20* in vitro. The pLVX lentiviral expression vector containing mouse *Krox20* cDNA and IRES-ZsGreen1 and its corresponding control vector, which expresses only ZsGreen, were used for transfection to overexpress *Krox20* in HEK293T and human hair follicular keratinocyte (HHFK) cells.

**Figure 7:**
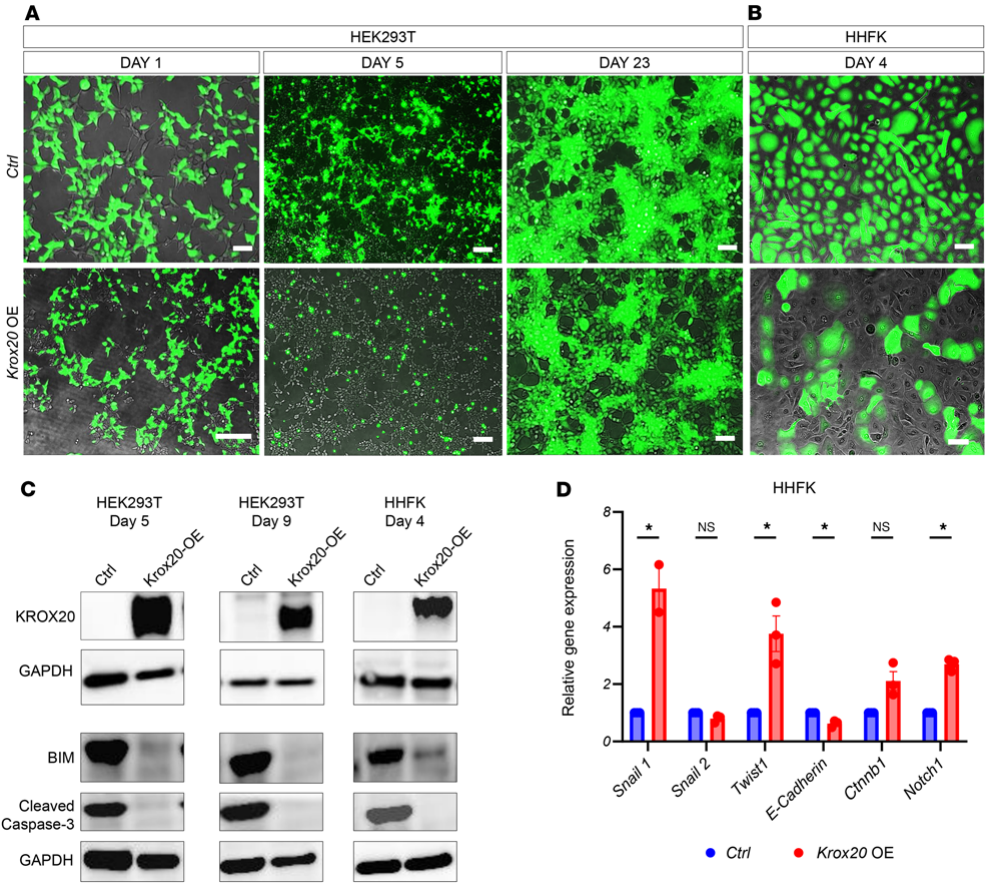
*Krox20* overexpression in vitro alters cell identity. Overexpression of *Krox20* (*Krox20*-OE) induces morphological changes in HEK293T cells (**A**) and a less pronounced change in HHFK cells (**B**). (**C**) Western blot analysis demonstrates downregulation of apoptotic markers in *Krox20*-OE cells. The same biological samples were run on a separate gel and probed for KROX20 and GAPDH. (**D**) qPCR demonstrates that overexpression of *Krox20* results in upregulation of EMT regulators (*Snail1*, *Twist1*, and *Notch1*) and downregulation of the epithelial marker *E-Cadherin*. Results are normalized to *GAPDH*. *n* = 3 replicates per condition. Data are shown as mean ± SEM. Two-tailed Student’s *t* test. *P <0.05. Scale bar: 100 μm.

In HEK293T cells, overexpression of *Krox20* caused morphological changes by 5 days after transfection, resulting in a more rounded morphology (Figure 7A). However, by 23 days after transfection, the overexpressing cells regained a normal morphology identical to that of the control cells (Figure 7A). However, due to the lack of proliferation in *Krox20*-overexpressing HHFKs, analysis of HHFK cells beyond 4 days after transfection was not possible (Figure 7B).

We hypothesized that the observed morphological change in HEK293T cells could indicate that the cells were undergoing either apoptosis or a change in cell identity. To assess apoptosis, we evaluated the levels of apoptotic markers BIM and Cleaved Caspase-3. Western blot analysis demonstrated a lower level of these apoptotic markers in *Krox20*-overexpressing HEK293T cells compared with their control counterparts (Figure 7C). Interestingly, *Krox20*-overexpressing HHFKs also caused downregulation of the apoptotic markers within 4 days after transfection (Figure 7C). To investigate if the cells were undergoing an identity change, we evaluated the expression level of various epithelial versus mesenchymal markers using quantitative PCR (qPCR) (Figure 7D). The *Krox20*-overexpressing HHFKs cells showed varying expression levels of these genes, including a significant upregulation of *Snail1*, *Twist1*, and *Notch1*, regulators of epithelial-mesenchymal transition (EMT) (23–26), and a downregulation of *E-Cadherin*, a marker of epithelial cells (27, 28) (Figure 7D). While these results do not establish a direct role for endogenous KROX20, they raise the possibility that *Krox20* overexpression may influence pathways involved in epithelial differentiation and survival, warranting further investigation in more physiologically relevant systems.

## Discussion

In this study, we showed that transcription factor KROX20 marks a population of stem cells expressed in the upper and middle HF that contribute to IFE. Interestingly, our research indicated that depletion of *Krox20*^+^ cells of the *K14* lineage leads to hyperplasia, marked by a clear expansion of *K14*^+^ cells, and aberrant differentiation/stratification of the IFE in adult mice (Figure 6). This suggests that *Krox20*-lineage cells play an important role in maintaining proper epidermal homeostasis. One potential explanation for the observed epidermal hyperplasia is the presence of other resident stem cell populations in the IFE that either counteract or overcompensate for the loss of *Krox20*-lineage cells.

Previous studies have demonstrated that HF stem cell populations can contribute to IFE regeneration, but this has mostly been observed under nonhomeostatic conditions (29–32). The most compelling evidence for the absence of HF lineage cells in the IFE comes from *Shh-* and *Sox9-*lineage tracing analyses (33, 34). In these studies, the majority of the HF is labeled, indicating the presence of lineage cells, while the IFE remains unlabeled. These findings, however, can be reconciled with our results. In the case of *Shh-*lineage analysis, a notable observation is the absence of these cells in the majority of the infundibulum (34), the dominant region of *Krox20* expression in the HF. This suggests that the unlabeled cells may actually represent *Krox20*^+^ cells with the capacity to regenerate the IFE. On the other hand, starting at approximately P8, *Sox9*-lineage cells encompass the majority of the HF, including the infundibulum (33), which likely includes *Krox20*^+^ and lineage cells. Notably, the reported lineage tracing in their study does not extend beyond P21, leaving open the possibility of subsequently detecting *Sox9*-lineage cells in the IFE during prolonged analyses. This observation would align with our results, wherein *Krox20*-lineage cells are not detected in the IFE until approximately P30–P40. Given the marked heterogeneity of *Krox20*-expressing cells observed in our scRNA-Seq data, we propose that not all *Krox20*-expressing cells possess the same degree of multipotency. It is conceivable that only a subset of *Krox20*^+^ cells contributes to the IFE and differs from those contributing to the HF. For this reason, labeling most cells with varying multipotencies at the onset of lineage tracing may more accurately determine the fate of this heterogeneous cell population. Conversely, conducting lineage tracing at the clonal level may not accurately reflect the full capabilities or characteristics of the broader cell population, even when multiple replicates are performed. This discrepancy may contribute to the differences observed in our lineage tracing results with *Krox20-CreERT* in this study compared with the *Lrig1-CreERT* line reported by Page et al. (4), despite the extensive overlap between these 2 stem cell populations (Figure 1C and Figure 4D). As part of our future research directions, we intend to differentiate between these distinct populations of *Krox20*^+^ cells through additional lineage tracing experiments.

Our findings hold important implications for future studies to understand mechanisms that regulate epidermal homeostasis. In addition, our findings set the stage to investigate the contributions of *Krox20*^+^ cells and their lineages in skin health and disease, with potential applications for skin repair following injury and burns.

## Methods

### Sex as a biological variable.

Our study examined male and female animals, and similar findings are reported for both sexes.

### Mice.

*Krox20-Cre* (35) (strain no. 025744), *K14-CreERT* (36) (strain no. 005107), *R26-tdTomato* (strain no. 007914), and *R26-YFP* (Strain no. 006148) mice were purchased from The Jackson Laboratory. *Krox20-flox-GFP-flox-DTA* mice (10) were a gift from Patrick Charnay (Mondor Institute for Biomedical Research, Creteil, France) and Piotr Topilko (Institut de Biologie de l’Ecole Normale Supérieure, Paris, France); *Krox20-flox-GFP-flox-DTA* functions as a knock-in allele, where GFP acts as a reporter for the *Krox20* promoter in the absence of Cre. When Cre is present, GFP is excised, and DTA is expressed to ablate the cells (10). In our study, we utilized *Krox20-GFP* to show the live expression of *Krox20*, while *Krox20-DTA* was employed when Cre was introduced to target and ablate *Krox20*^+^ cells. *Krox20-CreERT* mice were generated by the Children’s Research Institute (CRI) Transgenic Core at UT Southwestern.

### Immunostaining.

For immunofluorescence staining, frozen sections or paraffin sections after deparaffinization, rehydration, and antigen retrieval were used. The primary antibodies used in this study were: CD3 (B67, ab16669, Abcam), CD4 (ab183685, Abcam), CD8α (PA5-81344, Invitrogen), GFP/YFP (1020, Aves Labs), IBA1 (019-19741, Wako), CRE (NB100-56133, Novusbio), K14 (NBP234675B, biotin-labeled, Novus), K15 (ab52816, Abcam), Ki67 (15580, Abcam), KROX20 (PRB-236P, Covance; 27814, Invitrogen; or 13491-1-AP, Protein Tech), LOR (905101, BioLegend), and RFP/tdTomato (600-401-379S, Rockland). For immunofluorescence staining, the primary antibodies were visualized using secondary antibodies or streptavidin labeled with Cy3 or Alexa Fluor 488 (Jackson ImmunoResearch), with DAPI (Vector Labs) used for nuclei counterstaining.

### Microscopy.

Fluorescence microscopy images were captured using an Olympus fluorescence microscope (Model IX73) and the cell Sens Standard software (version 1.8). Image processing was conducted using Adobe Photoshop CS6 (version 13.0.1 x32), with adjustments limited to overall brightness/contrast and multicolor channel overlay. Statistical analysis was carried out using GraphPad Prism 8.

### Preparation of spatial transcriptomics libraries.

Tissue optimization for mice at P38 was performed using Visium Spatial Tissue Optimization Slide & Reagent Kit (PN-1000193) following the manufacturer’s protocol (document no. CG000238_VisiumSpatialGeneExpression). Fresh-frozen skin tissues from 3 *Krox20-CreERT; R26-tdTomato* mice at P38 were cut into 10 μm sections and placed on slides from the Visium Spatial Gene Expression Slide & Reagent kit (10X Genomics, PN-1000187), fixed, H&E stained, and imaged following the manufacturer’s instructions (document no. CG000160, 10X Genomics). Whole-slide digital images of full tissue sections were taken at 20× magnification using a Zeiss Axioscan.Z1 slide scanner in the Whole Brain Microscopy Facility at UT Southwestern Medical Center (RRID:SCR_017949), and images were visualized with Zeiss Zen Lite software (Blue version). Tissue permeabilization was performed for 12 minutes using Visium Spatial Tissue Optimization Slide & Reagent Kit (PN-1000193). Sequencing libraries were prepared following the manufacturer’s instructions (document no. CG000239_VisiumSpatialGeneExpression) followed by deep next-generation sequencing in the Microbiome Core Facility at UT Southwestern Medical Center. Novaseq6000 sequencer was used to sequence the samples and loaded on one lane of XP S4 Flow cells PE-150.

### Cell lines.

HEK293T cells were purchased from ATCC (catalog CRL-1573). Human hair follicular keratinocytes (HHFK) were purchased from ScienCell (2440, ScienCell).

### Krox20 overexpression.

A lentiviral vector was generated by cloning the mouse *Krox20* cDNA (MR227610L4, OriGene) into a pLVX lentiviral backbone that also contains IRES-ZsGreen1 (631982, Takara). Subsequently, HEK293T and HHFK cells were transfected with the lentiviral vector overexpressing mouse *Krox20*. Transfected cells were sorted by FACS for fluorescent cells.

### Western blot.

Cell lysates were subjected to Western blot analysis, using the following antibodies: KROX20 (13491-1-AP, Protein Tech); BIM (2933, Cell Signaling Technology); Cleaved Caspase-3 (9661, Cell Signaling Technology); and GAPDH (SC-32233, Santa Cruz Biotechnology Inc.).

### qPCR.

qPCR was performed on the various cells using the following primers (5′–3′): *Notch1*-Fwd: CACAACGAGGTCGGCTCCTA; *Notch1*-Rvs: ACAGTTCTGGCCGGTGAAG; *Ctnnb1*-Fwd: GACGGAGGAAGGTCTGAGGA; *Ctnnb1*-Rvs: TGGCCATGTCCAACTCCATC; *Snail1-*Fwd: TAATCCAGAGTTTACCTTCCAGCA; *Snail1-*Rvs: AGCCTTTCCCACTGTCCTCA; *Snail2-*Fwd: GCCAAACTACAGCGAACTGG; *Snail2-*Rvs: AGGAGGTGTCAGATGGAGGA; *Twist1-*Fwd: GGACAGTGATTCCCAGACGG; *Twist1-*Rvs: CATAGTGATGCCTTTCCTTTCAG; *E-Cadherin*-Fwd: CACCACGGGCTTGGATTTTG; *E-Cadherin*-Rvs: CAGCCAGTTGGCAGTGTCTC; *GAPDH*-Fwd: AGGGCTGCTTTTAACTCTGGT; and *GAPDH*-Rvs: CCCCACTTGATTTTGGAGGGA.

### 4-Hydroxytamoxifen and tamoxifen induction.

To induce *Krox20*-lineage cell depletion in *Krox20-DTA; K14-CreERT* mice, adult mice received tamoxifen (2 mg) orally once daily for 3 consecutive days. For lineage tracing with *Krox20-CreERT; R26-tdTomato* mice, adult mice were orally administered tamoxifen (1 mg) once daily for 3 consecutive days. Perinatal induction involved a single injection of 4-hydroxytamoxifen (40 μg) at the tail junction of 1- to 3-day-old pups.

### Genome-wide transcriptome analysis of Krox20 at the single-cell level.

Epithelial cells from epidermis of P5 *Krox20-GFP* mice were harvested following separation of the dermis from the epidermis by overnight incubation with dispase, as described in established protocols (37–39) and sorted by FACS for *GFP*^+^ (*Krox20*-expressing) cells using FACS Aria II SORP-5 lasers (PPMS for the Moody Foundation Flow Cytometry Facility at UTSW). Single-cell suspensions were loaded with Single Cell 3′ Gel Beads into a Next GEM Chip G and run on the Chromium Controller. Read 1 sequencing primer, complementary to the adapter region of the barcoded cDNA, was added during incubation. This primer initiates the first sequencing read, enabling capture of the cell barcode, UMI, and transcript sequence during subsequent library preparation and sequencing. Full-length barcoded cDNA was then amplified by PCR after cleanup. Sample size was checked and was then enzymatically fragmented and size selected before proceeding to library construction. After library preparation, quality control was performed using the DNA 1000 tape on the Agilent TapeStation 4200. Samples were loaded at 1.8 pM and run on the Illumina NextSeq500 High Output Flowcell using V2.5 chemistry.

### scRNA-Seq data analysis.

The analysis of single-cell transcriptomes was conducted using Cell Ranger 5.0.1 (10x Genomics, https://www.10xgenomics.com/). Raw sequencing data in BCL format were converted to FASTQ files and aligned to a custom mouse (mm10) reference transcriptome generated with cellranger mkref. Transcript counts for each cell were quantified using unique molecular identifiers and valid cell barcodes. The gene expression matrix from Cell Ranger was then input into the Seurat R package (v 4.0.5) for downstream analysis (40). Cells with fewer than 250 genes per cell and high mitochondrial gene content were filtered out. The LogNormalize method was used for global-scaling normalization. Highly variable genes were identified using the FindVariableFeatures module. The Shared Nearest Neighbor (SNN) graph was constructed with the FindNeighbors module by determining the k-nearest neighbors of each cell. Clusters were identified by optimizing SNN modularity using the FindClusters module, resulting in 10 clusters with a resolution of 0.6. Differential expression analysis, cluster visualization, and plotting were performed using Seurat.

### Statistics.

The statistical analysis was conducted using two-way ANOVA or unpaired 2-tailed Student’s *t* test, as specified in the figure legends (Prism8, GraphPad). The data are presented as mean ± SEM. A *P* value of less than 0.05 was considered statistically significant. Significant differences were denoted by asterisks (*P < 0.05; **P < 0.01; ***P < 0.001; ****P < 0.0001).

### Study approval.

Mouse care and experiments were approved by the IACUC at University of Texas Southwestern Medical Center and University of Virginia School of Medicine.

### Data availability.

All raw data are provided in the Supporting Data Values file. The single-cell RNA-Seq data and spatial transcriptomics data discussed in this publication have been deposited in NCBI’s Gene Expression Omnibus database, GSE281669 and GSE281086, respectively. Values for relevant figures are included with this manuscript as a Supporting Data Values file.

## Author contributions

LQL conceived and designed this research; EG, ET, TS, ZC, and YZ performed experiments; LQL, CPL, EG, ET, ZY, and CX analyzed data; EG and RMM wrote the paper; LQL oversaw the entire study. All authors read and approved the manuscript.

## Supplementary Material

Supplemental data

Unedited blot and gel images

Supporting data values

## Version 1 07/08/2025

In-Press Preview

## Version 2 08/22/2025

Electronic publication
