# Supplementary material for: Hair follicle epithelial stem cells contribute to interfollicular epidermis during homeostasis
Source: JCI Insight. 2025 Jul 8;10(16):e193496. doi: 10.1172/jci.insight.193496 (PMC12406716; doi:10.1172/jci.insight.193496)
Supplement: Supplemental data [file jciinsight-10-193496-s134.pdf]

Supplemental Figures:

Hair Follicle Epithelial Stem Cells Contribute to Interfollicular Epidermis during Homeostasis

Elnaz Ghotbi[1,#], Edem Tchegnon[1,#], Ze Yu[2], Tracey Shipman[1], Zhiguo Chen[3], Yumeng Zhang[3], Renee M. McKay[3], Chao Xing[2,4,5], Chung-Ping Liao[1,6], and Lu Q. Le[1,3,*]

*Author for Correspondence:

Lu Q. Le, MD, PhD
Professor and Chair
Department of Dermatology
University of Virginia School of Medicine
Charlottesville, VA, USA
Email: bkn6qd@uvahealth.org

Supplemental Figure 1

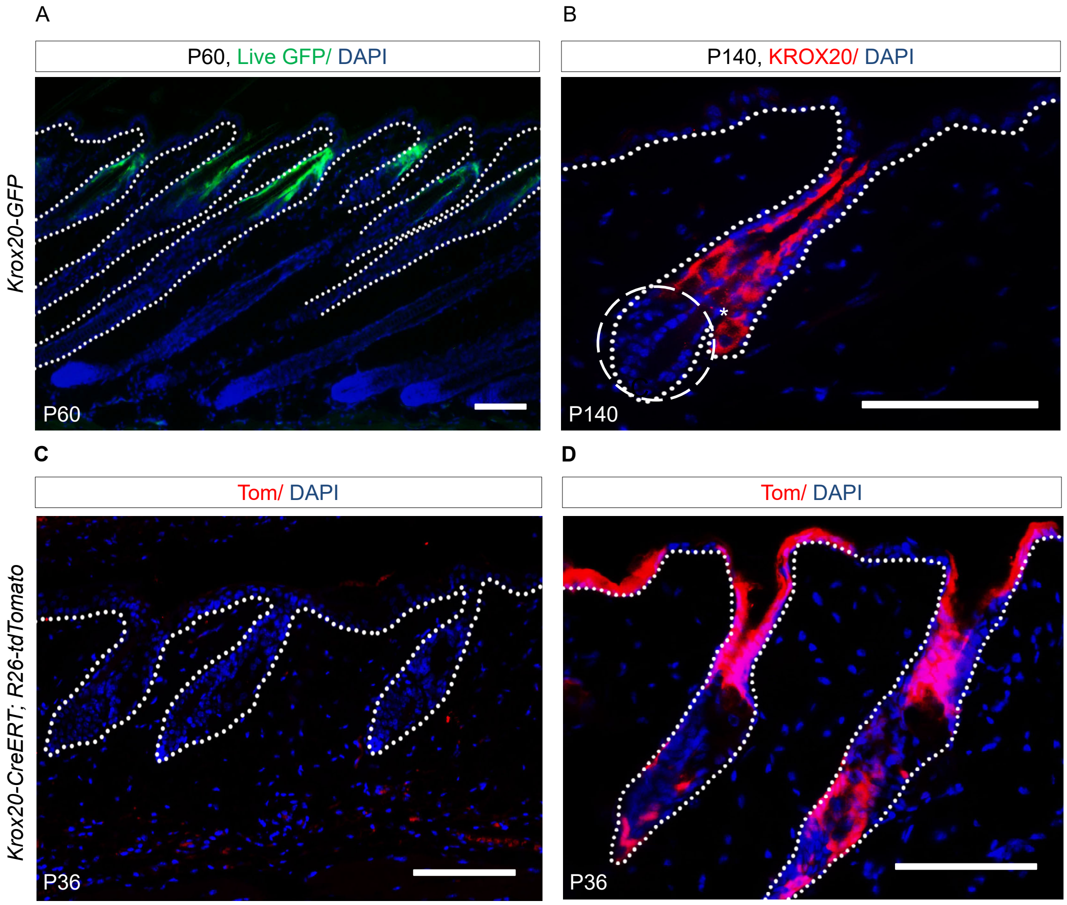

Supplemental Figure 1. KROX20 is a marker of stem cell populations in the upper and middle portions of the HF. Live GFP expression **(A)** and KROX20 antibody staining **(B)** in the dorsal skin of *Krox20-GFP* mice at P60 and P140, respectively, shows its restriction to the upper and middle HFs. Absence of tdTomato from the skin of *Krox20-CreERT; R26-tdTomato* mice at P36 indicates the absence of tamoxifen-independent recombination in these mice **(C)**, while P36 mice of the same genotype induced at P3 show labeling of most of the HF and IFE **(D)**. The asterisk represents the sebaceous glands. The dashed line circle represents the bulge area. $n \geq 5$. Scale bar, 100 μm.

Supplemental Figure 2

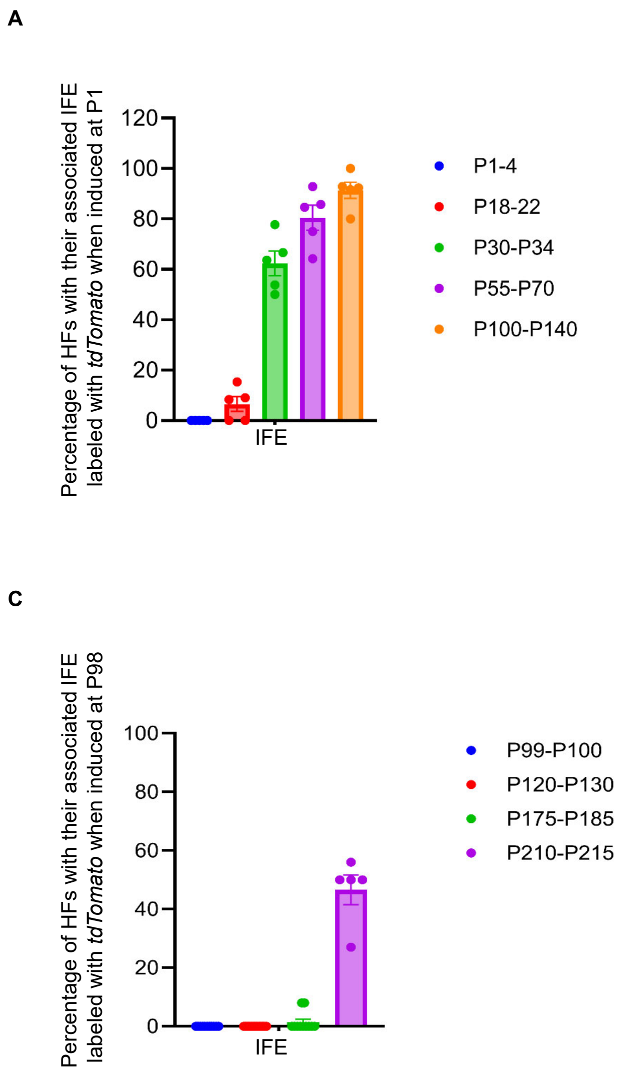

Supplemental Figure 2. **Quantification of the lineage tracings shown in Figure 2**. Quantification of HFs with their associated IFE labeled with *tdTomato* (*Krox20*-lineage cells) in *Krox20-CreERT; R26-tdTomato* mice when induced by tamoxifen at P1 (**A**), P55 (**B**) and P98 (**C**). $n \geq 4$.

Supplemental Figure 3

A

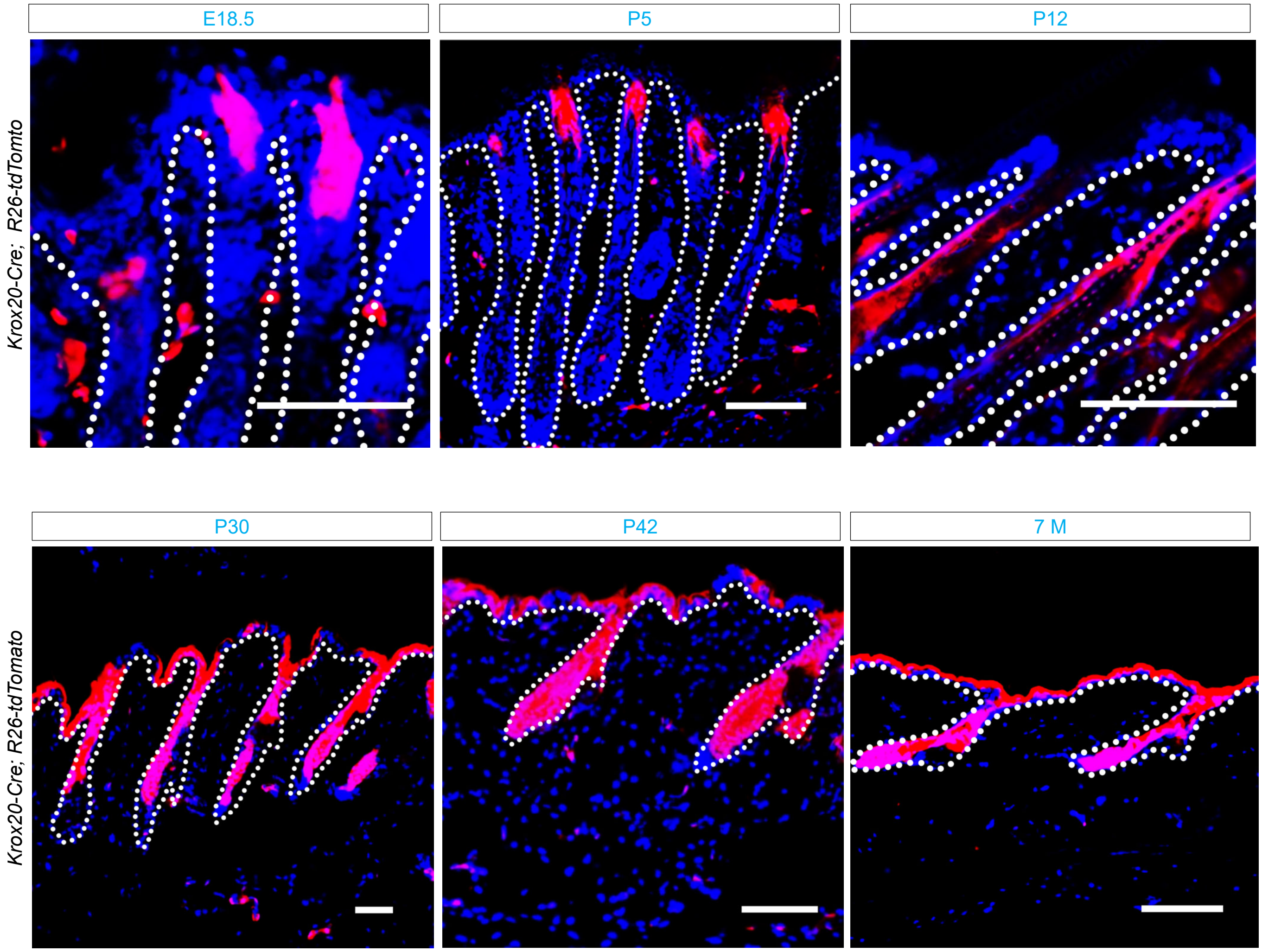

B

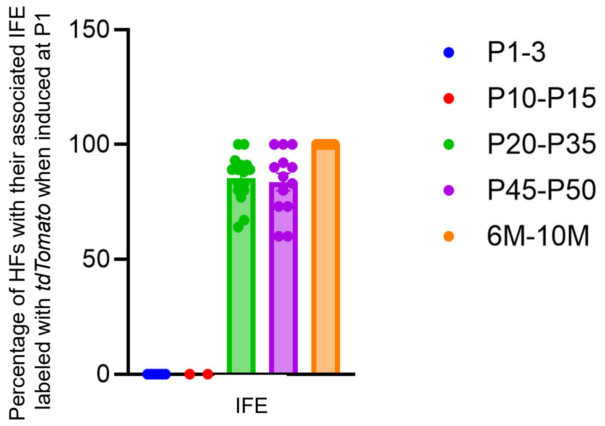

Supplemental Figure 3. Lineage tracing using *Krox20-Cre; R26-tdTomato* mice. (**A**) Lineage tracing of *Krox20* shows dynamic distribution of *Krox20*-lineage cells during mouse development. (**B**) Quantification of HFs with their associated IFE labeled with *tdTomato* in *Krox20-Cre; R26-tdTomato* mice. n ≥ 3. Scale bar, 100 µm.

Supplemental Figure 4

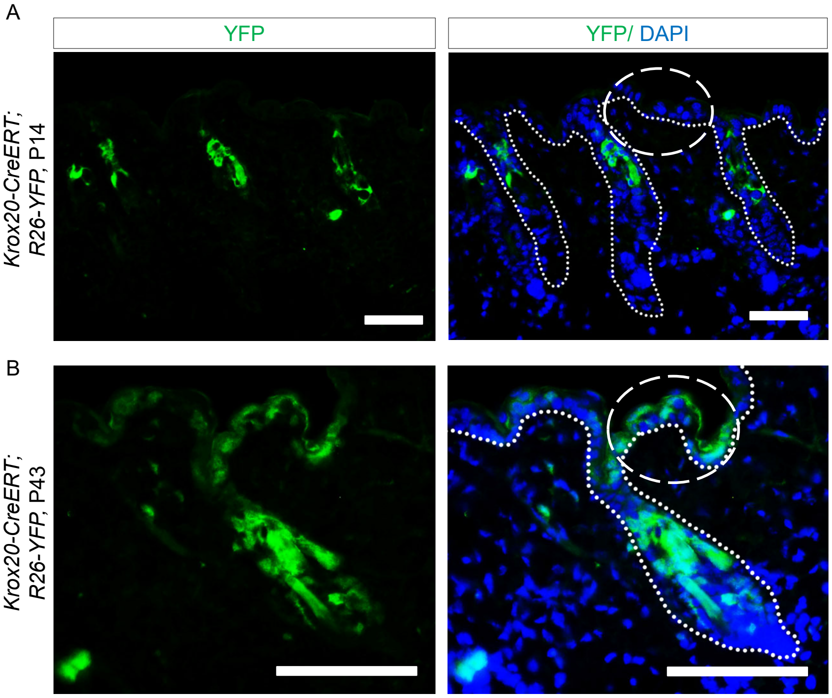

Supplemental Figure 4. Lineage tracing with *Krox20-CreERT; R26-YFP* confirms the contribution of *Krox20*-lineage cells to IFE. (**A, B**) Lineage tracing experiments were conducted using *Krox20-CreERT; R26-YFP* mice, where tamoxifen induction was performed at P3. The dorsal skin of mice was assessed at 14 days, (**A**) and 43 days (**B**) post-induction. (**A**) At 14 days post-induction, *Krox20*-lineage cells were observed to be restricted within the live *Krox20* expression domain, demonstrating limited migration. The dashed line circle highlights lack of YFP signal in the IFE. (**B**) By 43 days post-induction, *Krox20*-lineage cells were observed to have contributed to the IFE (dashed line circle). n ≥ 3. Scale bar, 100 µm.

Supplemental Figure 5

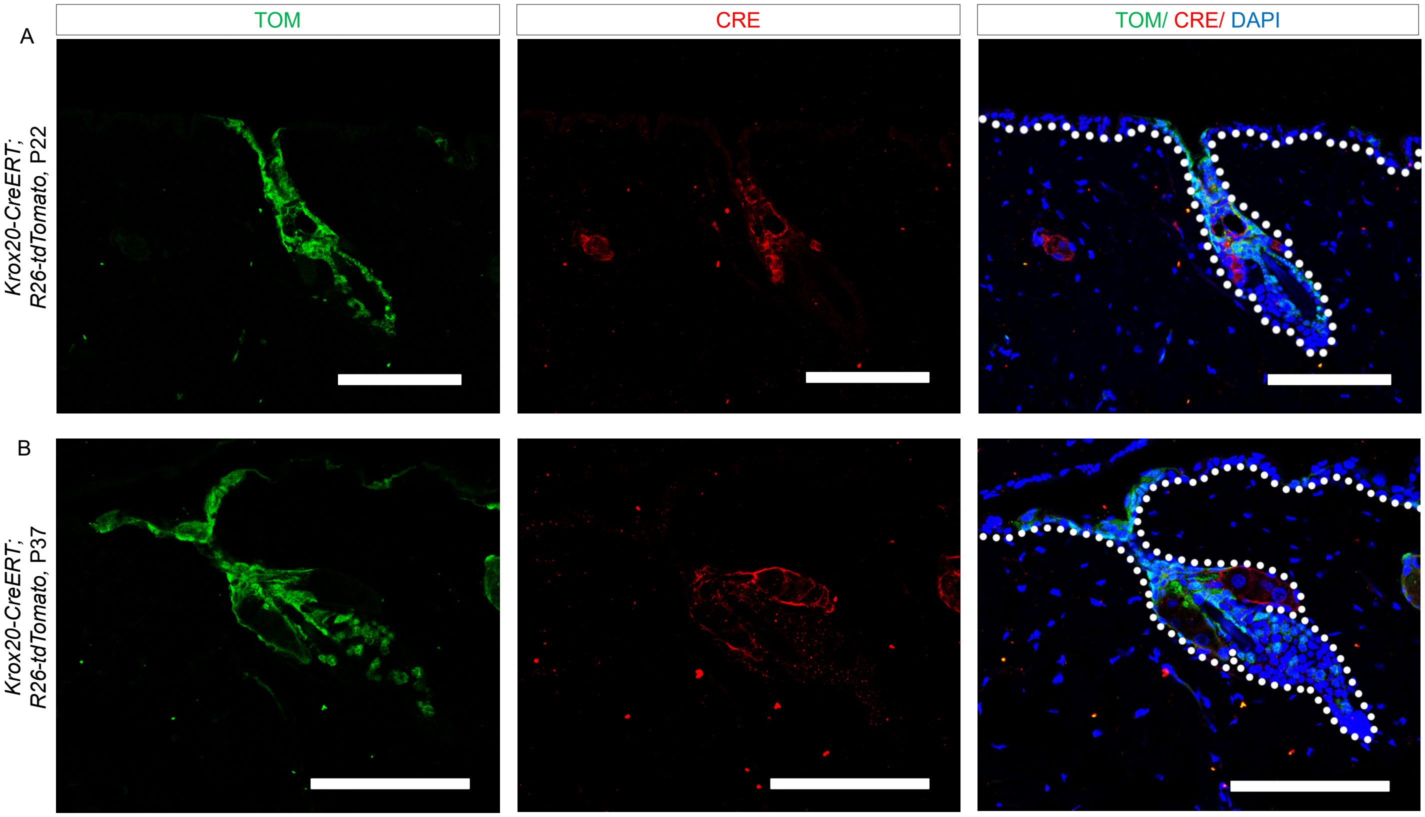

Supplemental Figure 5. Analysis of CRE expression in the skin of *Krox20-CreERT; R26-tdTomato* mice. (**A, B**) Skin analysis of *Krox20-CreERT; R26-tdTomato* mice induced with tamoxifen at P1 and analyzed at P22 (**A**) and P37 (**B**) showed that CRE expression was not detected in the IFE. n = 3. Scale bar, 100 μm.

Supplemental Figure 6

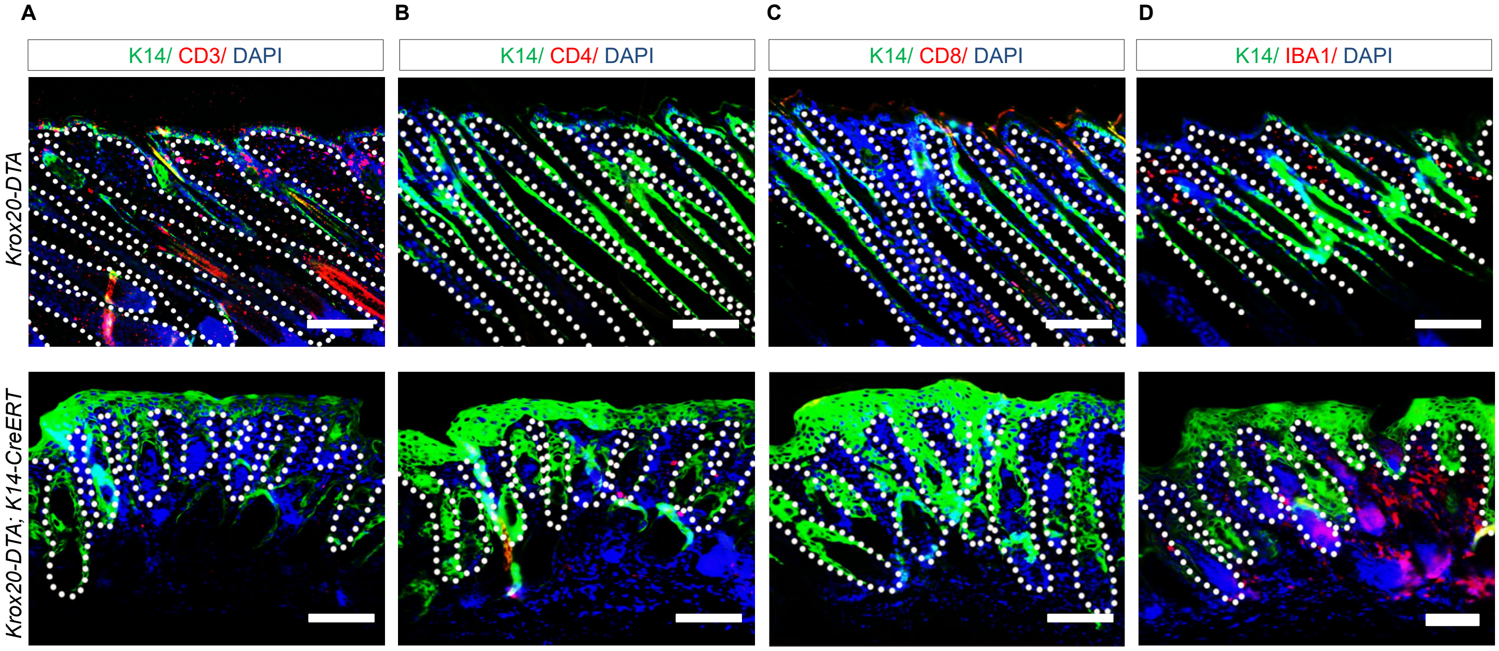

Supplemental Figure 6. Loss of epithelial *Krox20*-positive cells leads to epidermal thickening accompanied by elevated macrophage presence in the underlying dermis. Immunofluorescence staining of skin sections from *Krox20-DTA; K14-CreERT* mice at P41 induced at P32 revealed no notable increase in dermal infiltration of T cells ($CD3^+$, **A**), T helper cells ($CD4^+$, **B**), or cytotoxic T cells ($CD8^+$, **C**). In contrast, there was a clear increase in the number of macrophages ($IBA1^+$), within the dermal compartment (**D**). n = 3 mice. Scale bar, 100 µm.
