# Supplementary material for: Hair follicle epithelial stem cells contribute to interfollicular epidermis during homeostasis
Source: JCI Insight. 2025 Jul 8;10(16):e193496. doi: 10.1172/jci.insight.193496 (PMC12406716; doi:10.1172/jci.insight.193496)

Unedited Blots and Gel Images for:

Hair Follicle Epithelial Stem Cells Contribute to Interfollicular Epidermis during Homeostasis

Elnaz Ghotbi[1,#], Edem Tchegnon[1,#], Ze Yu[2], Tracey Shipman[1], Zhiguo Chen[3], Yumeng Zhang[3], Renee M. McKay[3], Chao Xing[2,4,5], Chung-Ping Liao[1,6], and Lu Q. Le[1,3,*]

[1]Department of Dermatology, University of Texas Southwestern Medical Center, Dallas, TX 75390, USA
[2]McDermott Center for Human Growth and Development, University of Texas Southwestern Medical Center, Dallas, TX 75390, USA
[3]Department of Dermatology, University of Virginia, Charlottesville, VA, 22903
[4]Department of Bioinformatics, University of Texas Southwestern Medical Center, Dallas, TX 75390, USA
[5]O'Donnell School of Public Health, University of Texas Southwestern Medical Center, Dallas, TX 75390, USA
[6]Graduate Institute of Medical Sciences, College of Medicine, Taipei Medical University, Taipei, Taiwan

# These authors contributed equally

*Author for Correspondence:
Lu Q. Le, MD, PhD
Professor and Chair
Department of Dermatology
University of Virginia School of Medicine
Charlottesville, VA, USA
Email: bkn6qd@uvahealth.org

Running title: Epithelial Stem Cell Population for Interfollicular Epidermis

Related to Figure 7C

HEK293T (DAY 5)

BIM

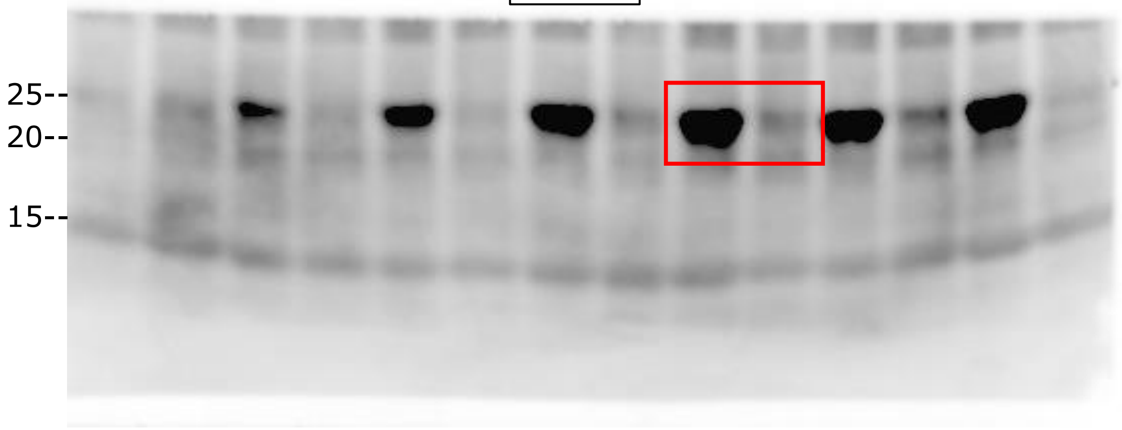

Cleaved Caspase-3

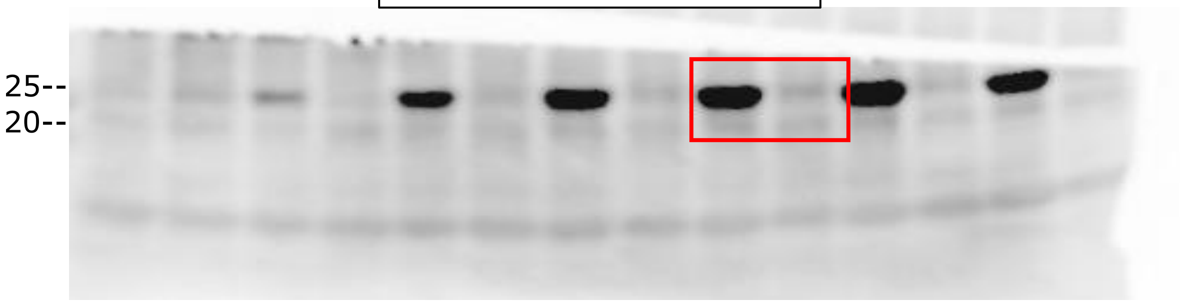

GAPDH

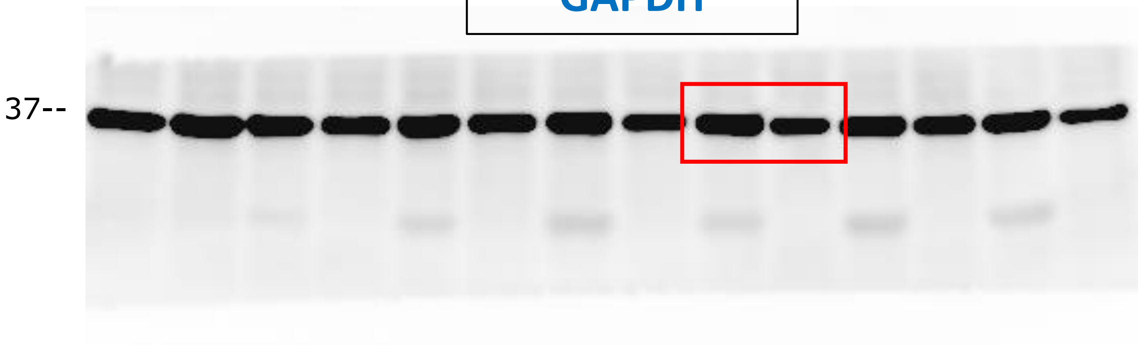

Related to Figure 7C

HEK293T (DAY 5)

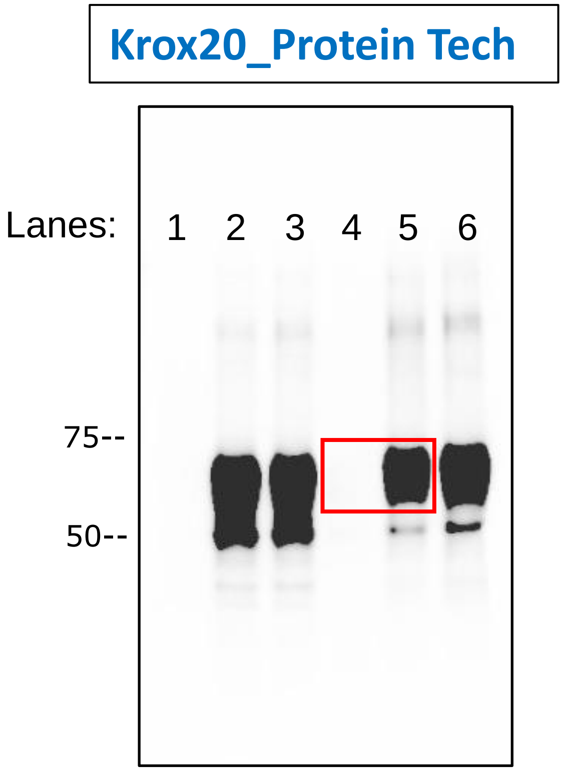

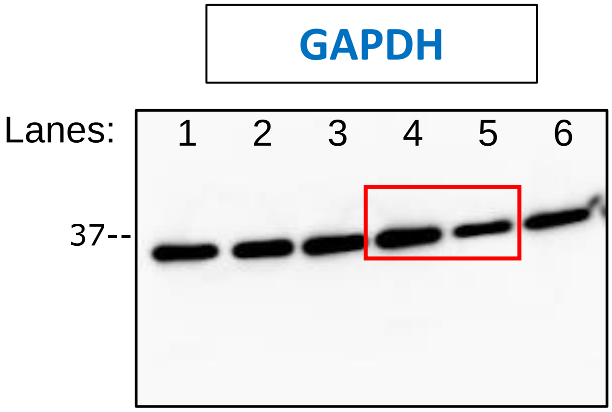

Related to Figure 7C

HEK293T (DAY 9)

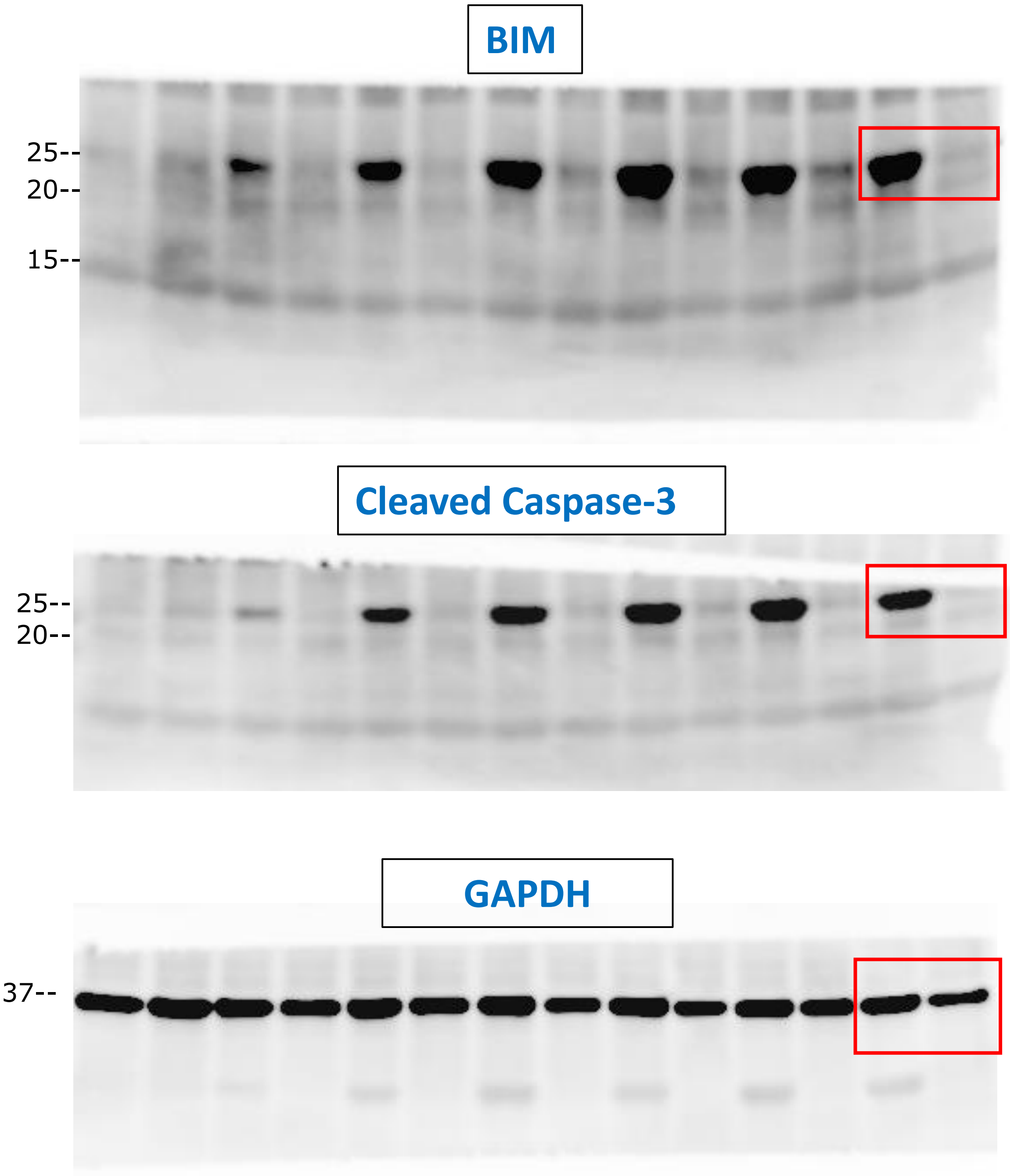

Related to Figure 7C

HEK293T (DAY 9)

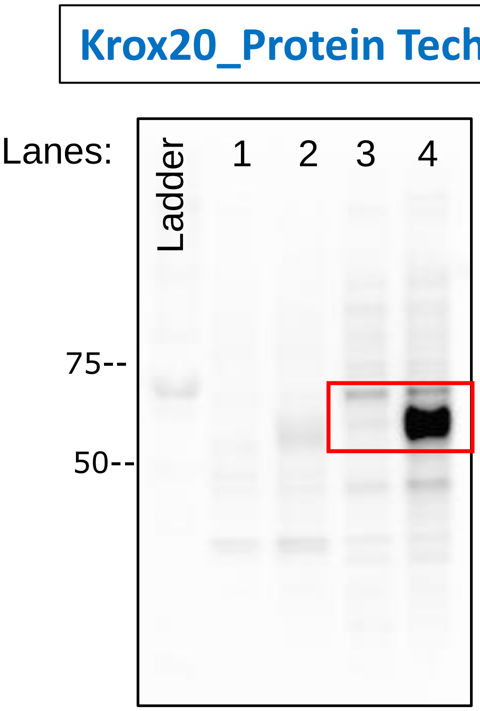

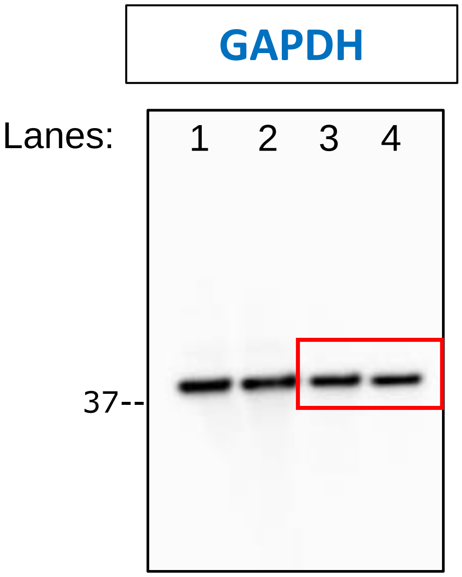

Related to Figure 7C

HHFK (DAY 4)

BIM

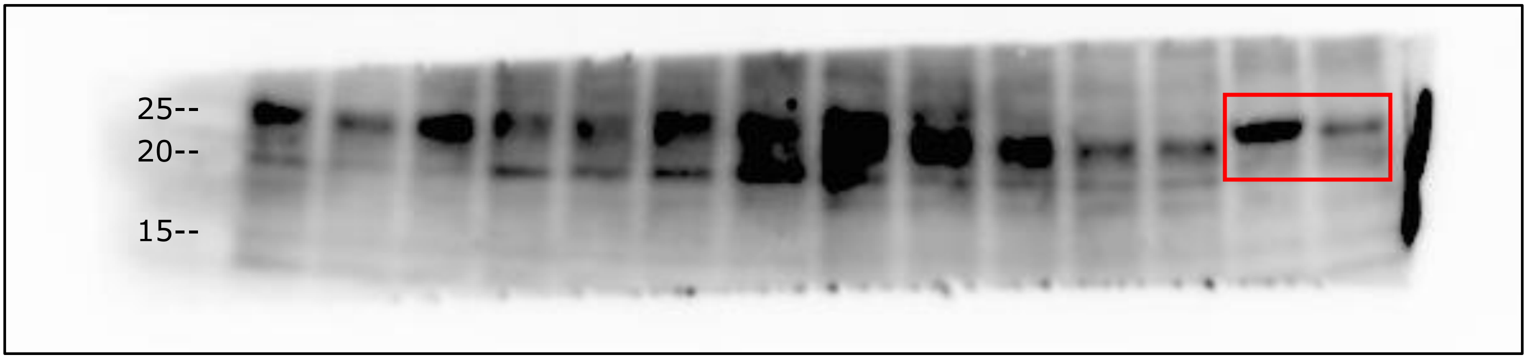

Cleaved Caspase-3

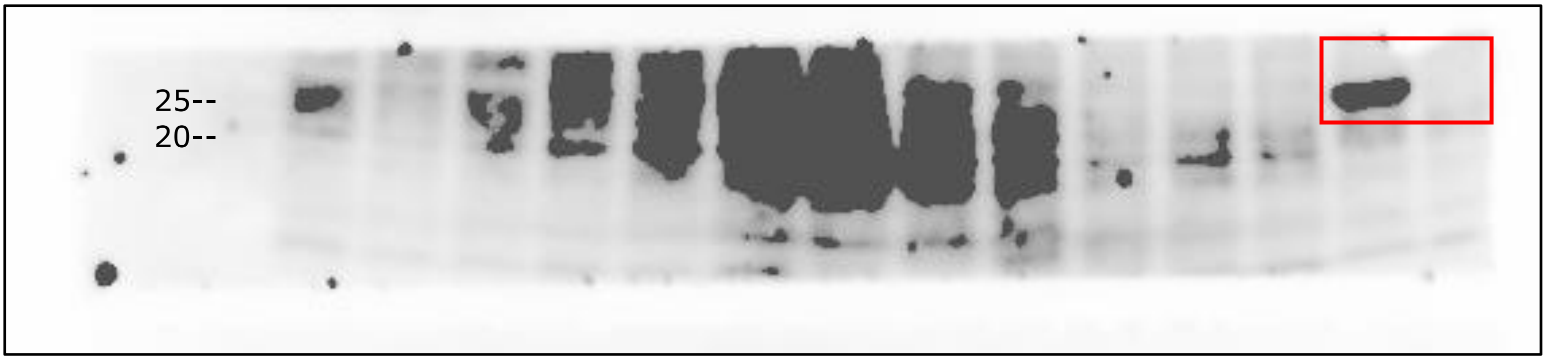

GAPDH

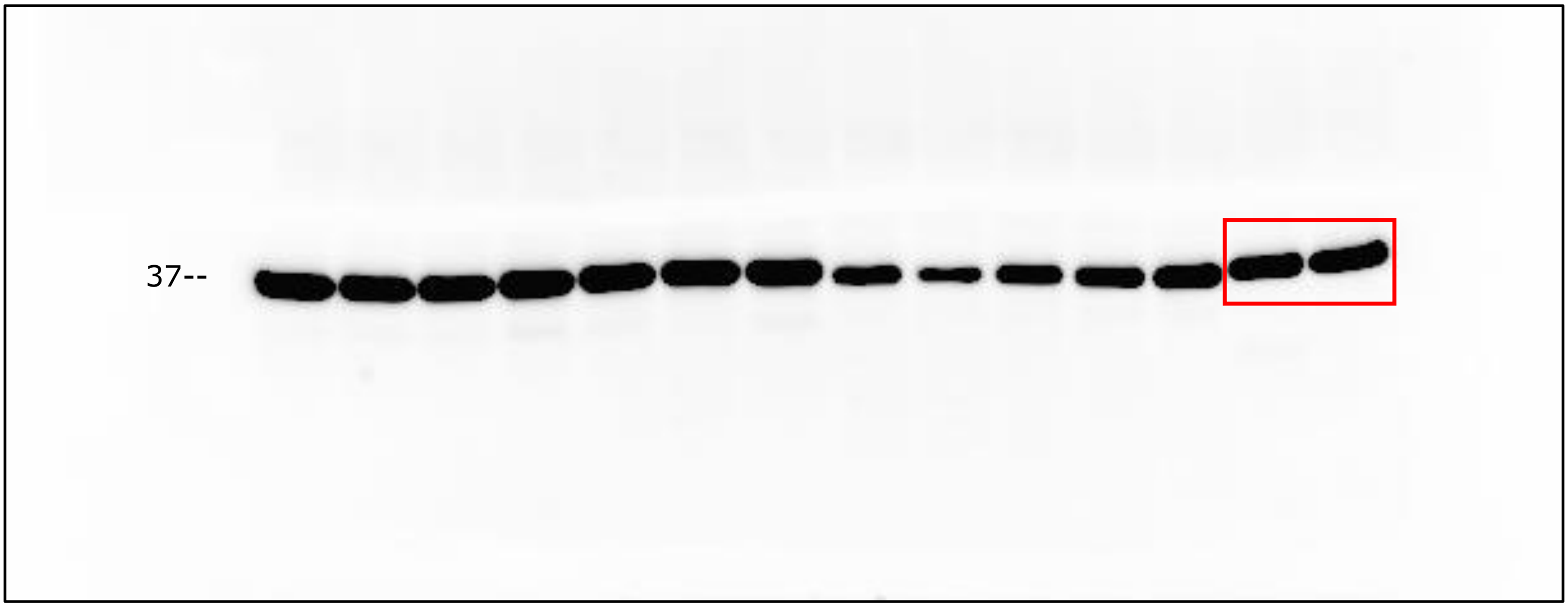

Related to Figure 7C

HHFK (DAY 4)

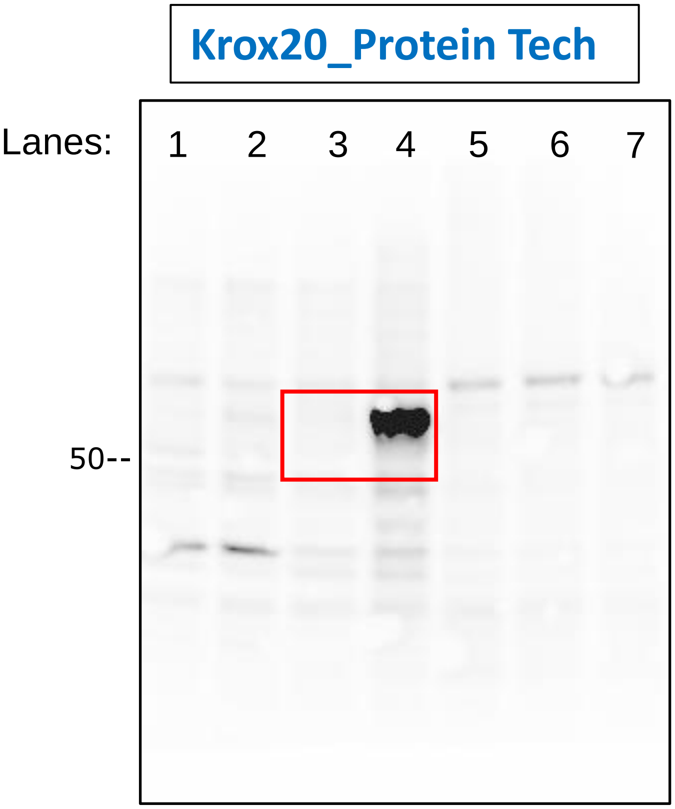

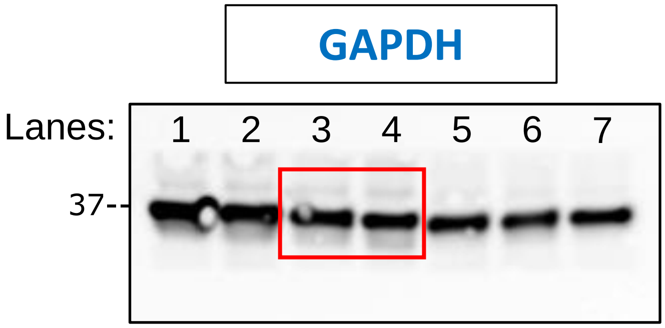

Supplement: Unedited blot and gel images [file jciinsight-10-193496-s135.pdf]
